# Unraveling Regulatory Programs for NF-kappaB, p53 and MicroRNAs in Head and Neck Squamous Cell Carcinoma

**DOI:** 10.1371/journal.pone.0073656

**Published:** 2013-09-19

**Authors:** Bin Yan, Huai Li, Xinping Yang, Jiaofang Shao, Minyoung Jang, Daogang Guan, Sige Zou, Carter Van Waes, Zhong Chen, Ming Zhan

**Affiliations:** 1 Department of Biology, Hong Kong Baptist University, Kowloon, Hong Kong; 2 Translational Gerontology Branch, National Institute on Aging, National Institutes of Health, Baltimore, Maryland, United States of America; 3 Head and Neck Surgery Branch, National Institute on Deafness and Communication Disorder, National Institutes of Health, Bethesda, Maryland, United States of America; 4 Clinical Research Training Program, sponsored by National Institutes of Health and Pfizer, Bethesda, Maryland, United States of America; 5 Methodist Hospital Research Institute, Weill Cornell Medical College, Houston, Texas, United States of America; National Institute of Genomic Medicine, Mexico

## Abstract

In head and neck squamous cell carcinoma (HNSCC), mutations of p53 usually coexist with aberrant activation of NF-kappaB (NF-κB), other transcription factors and microRNAs, which promote tumor pathogenesis. However, how these factors and microRNAs interact to globally modulate gene expression and mediate oncogenesis is not fully understood. We devised a novel bioinformatics method to uncover interactive relationships between transcription factors or microRNAs and genes. This approach is based on matrix decomposition modeling under the joint constraints of sparseness and regulator-target connectivity, and able to integrate gene expression profiling and binding data of regulators. We employed this method to infer the gene regulatory networks in HNSCC. We found that the majority of the predicted p53 targets overlapped with those for NF-κB, suggesting that the two transcription factors exert a concerted modulation on regulatory programs in tumor cells. We further investigated the interrelationships of p53 and NF-κB with five additional transcription factors, AP1, CEBPB, EGR1, SP1 and STAT3, and microRNAs mir21 and mir34ac. The resulting gene networks indicate that interactions among NF-κB, p53, and the two miRNAs likely regulate progression of HNSCC. We experimentally validated our findings by determining expression of the predicted NF-κB and p53 target genes by siRNA knock down, and by examining p53 binding activity on promoters of predicted target genes in the tumor cell lines. Our results elucidating the cross-regulations among NF-κB, p53, and microRNAs provide insights into the complex regulatory mechanisms underlying HNSCC, and shows an efficient approach to inferring gene regulatory programs in biological complex systems.

## Introduction

Transcriptional regulation of genes is governed by a combinatorial operation of multiple transcription factors (TFs) and microRNAs (miRNAs) at transcriptional and post-transcriptional levels. These factors can organize regulatory modules and thereby control expression of a set of genes in networks that carry out a variety of functional processes. Therefore, identification of regulatory modules and gene networks is critical for understanding molecular mechanisms of transcriptional regulation in complex biological systems. Various mathematical algorithms or computational methods have been developed for integrative analysis of microarray gene expression and TF binding data to predict target genes of TFs, such as Bayesian hierarchical network [1], Bayesian multivariate modeling [2], matrix decomposition [3] and regression model [4]. Based on predicted target genes of multiple TFs, we can unravel transcriptional regulatory modules and reconstruct gene networks. Among these methods, matrix decomposition was demonstrated to dissect regulatory relationships between TFs and genes in biologically complex systems. Statistically, this is a typical sparse matrix decomposition problem [5]. Several matrix decomposition methods, such as probabilistic sparse matrix factorization (PSMF), ModulePro and non-negative matrix factorization (NMF) have been implemented for regulatory network reconstruction based on the constraints of sparseness, non-negativeness, or partial network connectivity information [3], [6], [7]. Although these methods show improved results in uncovering biologically meaningful regulatory programs than the decomposition methods without these constraints, they are typically utilized separately, and no integrative framework has been utilized to bring the sparseness and pre-knowledge of regulator-target interactions together during matrix decomposition [3], [6], [8]. Here, we devised an integrative methodology, based on regulatory component analysis modeling, for inferring gene regulatory networks and uncovering transcriptional modules. The model-based method performs matrix decomposition under the joint constraints of sparseness and information of regulator-target connectivity, and allows an integrative analysis of gene expression profile and regulator binding data. In this method, the activity profiles of TFs or miRNAs are first constructed from the expression profiles of their target genes. The regulatory components are then derived by projecting gene expression data onto a sparse space of the regulator activity profiles, which should reveal quantitative relationships for regulatory network reconstruction and transcriptional module discovery. The clustering of TFs or miRNAs based on the regulatory components provides further clues for combinational roles of these regulators important for condition-specific gene regulation.

Here we utilized this newly developed method to analyze the complex regulatory networks of HNSCC. HNSCC represents 95% of head and neck cancers and is the sixth most common malignant tumor worldwide [9]. The development of HNSCC is associated with aberrant gene expression, which leads to diverse phenotypic alterations and could be regulated by multiple TFs or miRNAs. Among them, p53 and NF-κB play critical roles to modulate cellular proliferation, apoptosis, proinflammation, and therapeutic resistance [10], [11], [12]. As a tumor suppressor, p53 is implicated as a master regulator of apoptosis, cell cycle and DNA repair [13]. Mutations of p53 gene *TP53* have been observed in more than 40–50% of human solid tumors, including HNSCC [14]. Our previous promoter analysis has revealed a reciprocal relationship between p53 and NF-κB with two distinct over-expressed gene clusters in HNSCC [15]. In a systems biology study, we have also identified 748 potential NF-κB target genes that are functionally associated with HNSCC by using an integrative model COGRIM [16]. NF-κB and related signaling pathways have served as potential biomarkers and therapeutic targets for HNSCC and other human cancers [17], [18], [19]. Together with investigations from our and other laboratories, we have shown that p53 and NF-κB are critical regulatory determinants of multiple gene expression programs, interacting pathways, and malignant phenotypes of HNSCC [15], [20], [21]. In addition, other cancer-related TFs, such as AP1, STAT3, EGR1, CEBPB and SP1, have been experimentally and individually studied in HNSCC and other cancers [15], [22], [23], [24], [25], [26], and implicated in complex cross-talk with p53 or NF-κB pathways [22], [26], [27], [28], [29], [30]. However, the global modulation of gene expression by these TFs that control development and progression of HNSCC is not adequately understood. Moreover, alterations of miRNAs, such as oncogenic mir21, are well-documented for targeting p53 and NF-κB pathways [31], [32]. Tumor suppressor mir34 family (consisting of a, b and c) are direct transcriptional targets of p53 [33]. It remains unclear whether the altered miRNAs play as co-regulators to interact with transcriptional modulation by TFs, especially NF-κB and p53 in HNSCC.

In this study, we applied the newly developed method to identify transcriptional and post-transcriptional regulatory programs by combining TF or miRNA binding data and differentially expressed gene profiling in HNSCC cells. Our studies demonstrate that two master TFs NF-κB and p53 have a substantial impact on expression profiles of gene programs, including previously known NF-κB or p53 targets, or newly predicted ones. In addition, we defined two broader transcriptional regulatory programs of seven key TFs, including NF-κB, p53, AP1, CEBPB, EGR1, SP1 and STAT3. Furthermore, we reverse engineered regulatory networks of NF-κB, p53, mir21 and mir34ac in HNSCC cell lines and tissues, respectively. Our findings indicate that NF-κB, p53 and the miRNAs form concerted regulatory modules in the progression and metastatic program of HNSCC. We validated the target genes of NF-κB and p53 identified by the model-based method by silencing of the two TFs and testing p53 binding activity on promoters. Our results not only unveil predominant roles of NF-κB and p53 transcriptional regulation in affecting the gene expression of different HNSCC phenotypes, but also provide a framework for future experimental analysis of miRNA regulatory functions.

## Materials and Methods

### Microarray datasets

The microarray data were from ten HNSCC (UM-SCC: University of Michigan series of HNSCC) cell lines and four human normal keratinocytes [15], [34]. Tumor lines were divided to two subgroups, the mutant (mt) p53 and wild type (wt) p53-deficient cells that contain intact wtp53 genotype but expression is very low [15], [34]. We identified differentially expressed genes among the human normal keratinocytes and the HNSCC subgroups that satisfied ≥2.0 fold change of gene expression of either subgroup with the mt p53 or the wt p53-deficient status when compared with gene expression in the normal keratinocytes. In addition, two gene expression microarray datasets of HNSCC metastatic and non-metastatic tissues (accession numbers GSE2280 and GSE2379) were retrieved from the Gene Expression Omnibus (GEO) database at the National Center for Biotechnology Information (NCBI).

### TF and miRNA binding data

Binding data of nine TFs (RelA, cRel, NFκB1, p53, AP1, CEBPB, EGR1, SP1 and STAT3) and two miRNAs (mir21 and mir34ac) were extracted from previous publications or predicted conserved binding sites (see Text S1 for the detailed information).

### The model-based method and application in HNSCC

We devised a novel method for identifying target genes of regulators and inferring gene regulatory networks. The method is based on regulatory component analysis modeling, performing matrix decomposition under the joint constraints of sparseness and partial information of regulator-target connectivity. It allows an integrated analysis of gene expression profiles with binding data of a set of regulators (such as TFs, miRNAs, etc.).

The newly developed method is a network structure-driven model for inferring gene regulatory networks and uncovering regulatory modules. Given a microarray data matrix 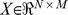 with the sample size *M* and the numbers of genes *N*, our aim is to find 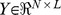 and 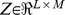 such that the square error (Euclidean distance) function:
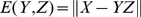
(1)is minimized under a desired degree of sparseness on the mixing matrix **Y**. We defined a sparseness measure 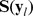 based on the relationship between the *L*_1_ norm and the *L*_2_ norm [35]:
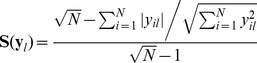
(2)where 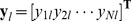 is the lth column of **Y**, and the superscript “**T**” means “transpose”. The L1 norm 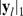 and the L2 norm 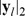 were defined as 
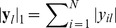
 and 
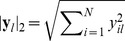
, respectively. The sparseness evaluates as one if, and only if 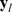 contains a single non-zero element, and takes a value of zero if and only if all elements are equal.

The rows of **Z** represent the expression profiles of the *L* latent variables across samples, and can also be viewed as the activity profiles of the *L* regulators. Thus, we can cluster genes based on corresponding non-zero coefficients of **Y**, which represent gene regulatory programs, i.e. regulatory modules that are co-regulated by the *L* regulators. In the new method, we developed an iterative learning algorithm that is capable of combining constraints of sparseness and limited information of regulator-target binding. The sparseness was used as a statistical parameter for modeling the regulatory components of TFs or miRNAs and their targets. The learning procedure was based on a projected gradient descent approach with sparseness constraints [35].

To run the model, two inputs are required: 1) microarray data matrix **X** and 2) regulator binding data matrix **C**. **C** can be constructed from Chromatin Immunoprecipitation (ChIP)-based (such as ChIP-seq and ChIP-chip) data, known regulator-target binding, or the promoter sequence analysis of conserved binding sites, etc. In general, there are five steps for the procedures:

- Step 1: Initial **Y** to a random matrix.
- Step 2: Construct **Z**. For inferring gene regulatory network, estimate **Z** from **X** and **C** based on the median of expression values.
- Step 3: Project each column of **Y** to have unchanged *L*_2_ norm, and *L*_1_ norm set to achieve a desired sparseness.
- Step 4: Update **Y** through iteration.
  - Set 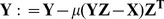, 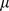 is the given step size.
  - Project each column of **Y** to have unchanged *L*_2_ norm, and *L*_1_ norm set to achieve a desired sparseness.
  - If 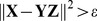, continue iteration; else stop.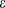 is the given convergence criterion.
- Step 5: Reconstruct regulatory network/gene modules. Module_*l*: =  (geneID [*i*], satisfy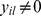, for 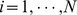 and 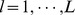). 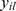 is the element of **Y** Module. *l* represents *l*th transcriptional module regulated by the TF *l* or the latent regulator *l*.

The output (**Y** matrix) of the procedure provides quantitative relationships between regulators (TFs and miRNAs in this study) and every gene from the microarray dataset. The non-zero values stand for regulatory interactions or components which can be used to estimate how possible a gene is regulated by the regulators or whether a gene is potential target of the regulators. To select the putative target genes, we re-evaluated **Y** matrix by including following criteria. We first performed 1000 runs by using randomly selected regulator-target binding data as inputs. The target genes of such regulator binding were randomly picked up from their corresponding microarray dataset during the model running. The 1000 randomly selected regulator binding data were used to construct **Z** matrices and generated new **Y** matrices following the same procedure, respectively. We then calculated *P* value of each gene in the original **Y** matrix created by using regulator binding data based on pre-knowledge or by promoter sequence analysis in comparison with the new **Y** matrices. In general, the majority of top genes in the original **Y** matrix have a lower *P* value. We selected genes with the top values or containing lower *P* values in the original **Y** matrix and identified them as putative target genes of TFs. The code of the model is available upon request.

We applied the model-based procedure to analyze HNSCC gene expression profile, TF and miRNA binding data, and reconstructed regulatory networks of HNSCC (see Table S1). The gene expression dataset (**X** matrix) was derived from microarray data of genes differentially expressed in HNSCC cells with the wt p53-deficient or the mt p53 status. We sought to unravel TF and TF-miRNA mediated regulatory networks responsible for the malignant phenotypes of HNSCC. Nine TFs were examined in this study: RelA, NFκB1, cRel, p53, AP1, CEBPB, EGR1, SP1 and STAT3, which were chosen because of their possible interactions with NF-κB family subunits or p53. In addition, we applied the model to analyze target genes of mir21 and mir34ac by integrating their binding data. Since the different tumor subgroups display distinct gene expression patterns cross all samples, we separated genes into four subsets: differentially over and under-expressed gene sets for wt p53-deficient and mt p53 subgroups, respectively. Through the analysis, we constructed four regulator-target binding activity matrices (**Z** matrix) for the regulators according to the corresponding gene expression profile of the subsets, respectively. Consequently, each **Y** matrix for TFs or miRNAs of the four gene subsets was calculated based on their corresponding **Z** matrix and expression data. Finally, we identified TF and miRNA regulatory modules which control different gene expression programs in HNSCC cell lines and tissues.

### Knocking down *RelA* and *TP53* by siRNA

The knockdown of *RelA* and *TP53* mRNA was performed by using siRNA (ON-TARGET plus SMARTpool; Dharmacon, Lafayette, CO). UM-SCC 1 (wt p53-deficient) and 22B (mt p53) cells were seeded in 6-well plates at 1×10^5^/well. At 50–60% confluency (24 h later), cells were transfected with 50 nM of a mixture of four siRNA oligos directed against human *RelA*, or *TP53* designed by Dharmacon, or 50 nM of a non-silencing control siRNA (QIAGEN, Valencia, CA), using 1∶200 Lipofectamine 2000 (Invitrogen, Carlsbad, CA) in Opti-MEM I Reduced Serum Medium (Invitrogen, Carlsbad, CA) for five hours. At 48 h or 72 h post-transfection, cells were harvested in Trizol for RNA isolation (Invitrogen, Carlsbad, CA).

### Real time RT-PCR

RNA isolation and cDNA synthesis were performed as previously described [36]. Real time PCR primers and probes for ΔNp63 [36] were synthesized by Applied Biosystems (Foster City, CA). Other primers and probes were purchased through Assays-on-Demand™ program from Applied Biosystems. Amplification conditions were: 2 min at 50°C and 10 min at 95°C, followed by 40 cycles of 15 sec at 95°C and 1 min at 60°C, carried out using an ABI Prism 7700 Sequence Detection System (Applied Biosystems). Relative gene expression values were calculated after normalization to 18S rRNA, and adjust the value of the control sample as 1. Each experiment was done in duplicates, and each sample was assayed in triplicates. Data were presented as mean + standard deviation (SD).

### p53 DNA binding assays

p53 DNA binding activity was quantitatively assessed using a modified version of the TransAM p53 ELISA kit from Active Motif (Carlsbad, CA). The regular oligonucleotide-coated 96-well plate was substituted with a streptavidin-coated plate and custom 5′-biotinylated double-stranded oligonucleotides containing known or putative p53 binding sites were added (IDT, Coralville, IA) to the assay. Each binding site was approximately 20–30 bp and flanked by 30–40 bp of native sequence upstream and 7 bp downstream. The sequences for the forward strand of each double-stranded oligonucleotide were as follows: *IL6*: 5′ biotin-TAGGCTTGGCGATGGGGTTGAAGGGCCCGGTGCGCATGCGTTCCCCTTGCCCCTGCGTGT -3′ (sequences underlined refer to core consensus binding sites of p53 predicted by Genomatix MatInspector); *SERPINE1*: 5′ biotin-ATTCTCCTGCCTCAGCCTCCCAAGCAGCTAGGATTACAGGCACGCACCACCATGCCTAGCTGATTT-3′; *MMP1*: 5′ biotin-ATTAACTCACCCTTGTTTCCCAGGCCTCAGTGGAGCTAGGCTTGTCACGTCTTCACAGTG -3′; *PLAU*: 5′ biotin- AGCTGCGGGCAAGGGGGTCTGAGGCAGTCTTAGGCAAGTTGGGGCCCAGCGGGGAGAAGT -3′; *CDKN1A* (p21): 5′biotin-GCAGGCTGTGGCTCTGATTGGCTTTCTGGCCGTCAGGAACATGTCCCAACATGTTGAGCTCTG -3′, where p53 binding site on *CDKN1A* promoter is known. Other reagents from the kit (buffers, antibodies, and substrates) were used as is.

Nuclear lysates containing p53 protein were obtained from UM-SCC 1 cells overexpressing p53. The cells were transfected with p53 plasmid using Lipofectamine, per the manufacturer's protocol (Invitrogen, Carlsbad, CA). After 48 hours, nuclear protein was isolated using a nuclear extract kit (Active Motif, Carlsbad, CA, USA). The protein concentration was determined using the BCA method (Pierce, Rockford, IL, USA). The 5 μg of nuclear extract in cell lysis buffer and complete binding buffer and 1 pmol of biotinylated oligonucleotide were combined and incubated for 30 min at room temperature prior to placement in a streptavidin-coated well. Specificity of binding was also tested using cold competition (unbiotinylated) oligonucleotides which contained the p53 consensus binding sequence (“wt competition”) or non-mammalian DNA (“mt competition”) at 40 pmol/well, added to the reaction at room temperature prior to plating. These competition oligonucleotides had a 10 bp core sequence flanked by 7 bp of non-specific sequence both upstream and downstream of the consensus motif. Nuclear extract from MCF-7 cells (a human breast adenocarcinoma cell line) (Active Motif) combined with a biotinylated oligonucleotide containing a known p53 binding site in the *CDKN1A* gene promoter (“MCF-7 p21 oligo”) served as a positive control (Active Motif). Wells containing only the “p21 oligo” biotinylated oligonucleotide and no cell lysate served as the negative control. Each binding site was assayed in duplicated. After developing the final HRP-mediated colorimetric reaction for 2–3 min, absorbance was measured at 450 nm by μQuant ELISA microplate reader (Bio-Tek, Winooski, VT, USA).

## Results

### The model-based approach to determine the interactions between TFs or miRNAs and target genes

We present a novel bioinformatics method for an integrative analysis of regulators (TFs and miRNAs) and gene expression profiles in HNSCC. An important feature of the method is its capacity to combine diverse data sources and perform matrix decomposition with sparseness constraint by regulatory component analysis. This integrated analysis offers an opportunity to infer regulatory components, which profile the interactions of each regulator with all the genes. The inferred interaction between regulators and their targets are condition specific, depending on the projection of condition specific expression data to the sparse and non-negative space of latent variables (regulators).

**Figure 1:**
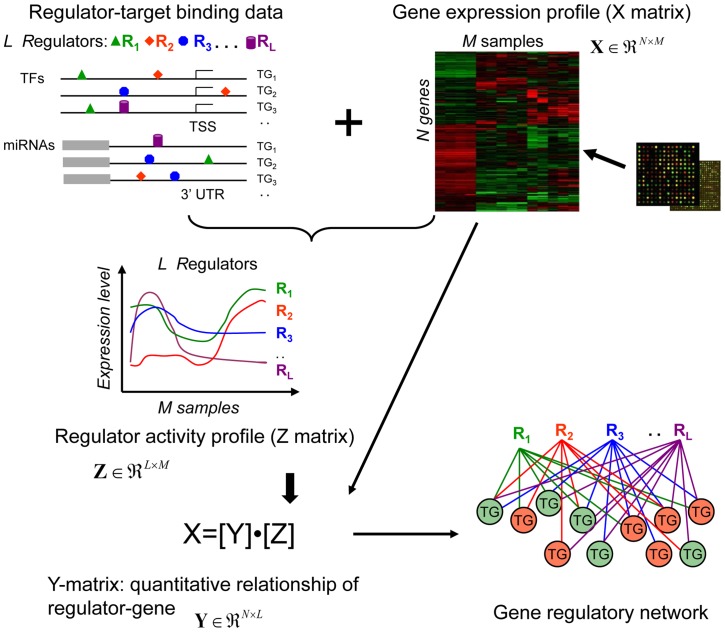
Overview of the regulatory component analysis model-based method. R: regulators (TFs, miRNAs). TG: target genes. TSS: transcriptional start site. 3′UTR: 3′ Translated region. Nodes in red and green represent up and down-regulated target genes, respectively.

Figure 1 outlines the approach based on regulatory component analysis modeling. First, two input data matrices are required: the microarray profile of gene expression (**X** matrix, *N* genes × *M* samples) and regulator-gene connectivity matrix (*N* genes × *L* regulators). The connectivity between a regulator (TF or miRNA) and a gene is set to 1 if there is binding between the regulator and the gene in the microarray datasets, or set to 0 otherwise. Second, the regulator activity profile (**Z** matrix, *L* regulators × *M* samples) of each regulator is constructed by integrating the expression profile and the regulator-gene connectivity. The regulator activity profile represents the expression pattern of each regulator across all samples, determined by the median of expression values. Third, the **Y** matrix (*N* genes × *L* regulators) is generated according to **[X]**  =  **[Y]⋅[Z]**, where contains quantitative relationships for each regulator-gene pair. Each column of **Y** matrix represents a regulatory component, derived by projecting gene expression data onto a sparse space of the regulator activity profiles. The **Y** matrix allows determining whether a gene is potential target of a regulator, i.e. TF or miRNA. Ideally, **Y** matrix can capture non-zero regulatory components between regulators and their target genes. Such regulatory components provide a basis for reconstructing regulatory networks through unsupervised learning, or can be used to cluster genes into regulatory programs or networks. Therefore, in the final step, such gene regulatory programs or networks are identified, and then classified to biological functions. The inferred programs or networks are sets of genes co-regulated by multiple TFs or miRNAs known or hidden.

Using the newly developed method, we first identified putative target genes of the nine TFs and their gene regulatory programs. We found that our method is able to dissect regulatory interactions between TFs and their target genes in HNSCC, and performed better when compared to the COGRIM method that was previously used [16]. The COGRIM method is based on a Bayesian hierarchical model and Gibbs sampling implementation, and predicts regulatory modules by an integrative analysis of microarray gene expression and TF binding motif data [1]. We compared our method to COGRIM to predict target genes by using the same microarray datasets based on a Gene Ontology (GO) analysis of the target genes of the three NF-κB subunits, RelA, NFκB1 and cRel. We assessed the functional relevance of GO biological processes based on the enrichment analysis by Fisher's exact tests. Table 1 shows the statistical enrichment of biological processes among the target genes identified by the two methods. The enrichment level was calculated by transforming the enrichment *P* values after False Discovery Rate (FDR) correction to negative log_10_ values and averaged over all biological processes with corrected *P<*0.05. If no functional modules were found with corrected *P<*0.05, the smallest value of corrected *P* was taken for calculating the enrichment level. Overall, our method showed a better performance than COGRIM. The averaged *P* values of over-representation and FDR values were lower than COGRIM in both wt p53-deficient and the mt p53 datasets. With the exception of NFκB1 targets in the wt p53-deficient dataset, our method in general displayed lower corrected *P* values of FDR (Table 1). The comparative analysis indicates that our method can lead to a more biologically meaningful prediction of TF target genes and regulatory programs.

**Table 1:** Comparison based on GO functional enrichment.

p53 type ^a^	TFs	Over-representation ^b^		FDR ^c^	
		Our method	COGRIM	Our method	COGRIM
wt	RelA	5.08	4.81	1.92	1.81
	NFκB1	3.89	4.04	1.66	1.7
	cRel	3.41	2.93	1.59	1.08
	Average of TFs	4.13	3.93	1.72	1.53
mt	RelA	3.86	3.09	1.8	0.89
	NFκB1	3.23	3.63	1.65	1.55
	cRel	3.69	3.29	1.74	1.42
	Average of TFs	3.59	3.34	1.73	1.29

All predicted target genes of three NF-κB subunits were used for GO functional analysis. The enrichment level was calculated by transforming enrichment *P* values averaged over all processes with False Discovery Rate (FDR) corrected *P*<0.05. If no processes are found with corrected *P*<0.05, the smallest value of corrected *P* was taken for calculating the enrichment level. ^a^ p53 type refers to subgroups of HNSCC cell lines divided by p53 mutation: wt p53-deficient and mt p53. ^b^ enrichment of Fisher exact *P* value after negative log_10_ transformation. ^c^ FDR correction to the negative log_10_ value.

### Prediction of HNSCC-specific target genes of TFs

**Figure 2:**
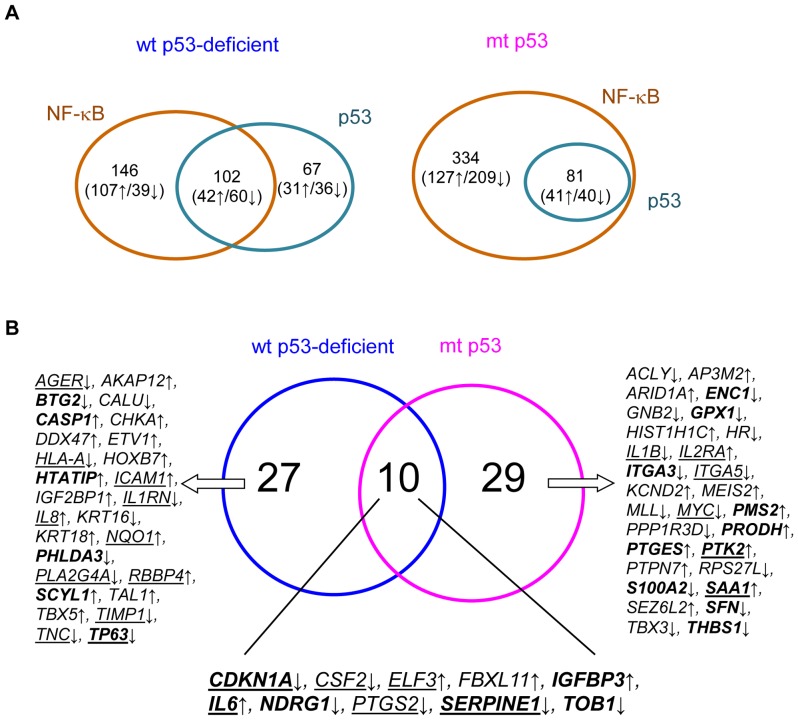
Transcriptional gene regulatory programs in the HNSCC cell lines. A, Overlapped target genes of NF-κB and p53. B, Gene regulatory programs of seven TFs (NF-κB, p53, AP1, CEBPB, EGR1, SP1, and STAT3). Genes in underlined, bold and bold-underlined refer to known targets of NF-κB, p53, and both NF-κB/p53, respectively. *↓* and *↑* refer to number of predicted target genes differentially over- and under-expressed in the tumor cells, respectively.

Our analysis predicted 248 target genes of NF-κB in the wt p53-deficient cells of HNSCC, which include 149 over- and 99 under-expressed genes (Figure 2A). These genes were putatively targeted by at least one of three NF-κB subunits, i.e. RelA, NFκB1 or cRel, representing 51% and 20% of differentially over and under-expressed subsets, respectively. Thus, a majority of the predicted NF-κB targets are up-regulated in the wt p53-deficient cells, consistent with the previous observation that NF-κB binding dominates the promoters of over-expressed genes in the same type of HNSCC cells [15], [34]. Among all the predicted target genes, 28 over- and 32 under-expressed genes were common targets of all the three NF-κB subunits. On the other hand, in the mt p53 cells, we predicted a set of 418 NF-κB targets, which represent 40% and 56% of the differentially over- and under-expressed gene sets, respectively (Figure 2A). A set of 62 genes were jointly targeted by the all three subunits of NF-κB, among which 19 genes were over- while 43 were under-expressed. Within all the predicted NF-κB target genes from the two tumor subgroups, 83 are identical to known NF-κB targets.

To discover HNSCC-specific p53 targets, we incorporated known binding data for p53 (see Materials and Methods) and the gene expression profiling data. The analysis identified 169 and 81 putative p53 target genes from the wt p53-deficient and the mt p53 subgroups, respectively (Figure 2A). The two subgroups shared 7 over- and 22 under-expressed target genes, indicating more common p53 genes are down-regulated in tumor cells. In total, 36 genes of the total predictions are consistent with known p53 targets, while others are novel candidates. These predicted p53 targets could be annotated to GO biological processes that are similar to those mediated by NF-κB gene programs.

To identify gene regulatory programs of multiple TFs, we first constructed two networks linking each TF and their putative targets predicted in the two tumor subgroups (Figure S1). A total of 298 and 232 genes were identified as common targets of at least two TFs in the wt p53-deficient and the mt p53 cells, respectively. We then compared target genes of NF-κB or p53 with other five TFs (AP1, CEBPB, EGR1, SP1 and STAT3). Table S2 shows the numbers of overlapped target genes. We detected significantly overlapped target genes between NF-κB or p53 and the other TFs in three gene subsets except for the over-expressed wt p53-deficient cells. In this subset, we detected significant overlaps of target genes between NF-κB or p53 and three TFs CEBPB, EGR1 and STAT3. Although not statistically significant, 53–58% of the target genes of p53, AP1, and SP1 were overlapped with the NF-κB targets. Our analyses provide evidence that NF-κB or p53 interact with the other five TFs to commonly regulate gene expression programs in HNSCC cells.

Notable was that all p53 target genes predicted in the mt p53 cells were also NF-κB targets (Figure 2A), whereas those overlapping in the wt p53-defficent cells was 60% (*P* <2.04×10^−18^, *R* = 1.9). Specifically, the intersection of target genes between p53 and the three NF-κB subunits was also significant in the same three gene subsets (Table S2). On the other hand, we noted that a fraction of the NF-κB target genes overlapped with p53 targets seems different between the wt and mt p53 subgroups. Among the total NF-κB target genes, 41% were overlapped with the p53 targets in the wt p53 deficient, which was greater than those in the mt p53 cells (19%). This difference is due to the different fractions of their overlapped genes observed in the under-expressed gene subsets (61% vs. 16%), suggesting that most of NF-κB down-regulated genes are targeted by p53 in the wt p53-deficient cells. We did not find such a difference in the over-expressed gene subsets (28% vs. 24%). Next, we annotated co-targets of NF-κB and p53 to gene ontology (GO) biological processes (Table S3A). The top enriched processes in the both wt and mt p53 cell lines included regulation of key functional processes important in cancer, such as proliferation, angiogenesis, apoptosis, inflammatory responses, and cell migration. Furthermore, we identified significantly enriched canonical signaling pathways (*P*<0.05) using the Ingenuity Pathway Analysis (IPA) tool. As shown in Table 2, fifteen pathways, including Ephrin receptor, ERK/MAPK, HGF, IGF-1, IL-6, IL-8, Integrin linked kinase, Integrin, Neuregulin, p38 MAPK, p53, PI3K/AKT, PPAR, PTEN, and TREM1 were enriched in the both tumor subgroups. Most genes in the pathways were down-regulated (Table S3B). *CDKN1A, IL6, PTK2, PTGS2* and *TP63* are known target genes of both NF-κB and p53.We also detected 14–3–3-mediated, HIF1α, Leukocyte extravasation, mTOR, NF-κB, TGFβ, VEGF, and Xenobiotic metabolism signaling enriched in the wt p53-deficient subgroup. The majority of genes in the eight pathways were also down-regulated (Table S3B). In contrast, four enriched pathways, CDK5, HER-2 in breast cancer, Protein kinase A, and Small cell lung cancer signaling, were observed in the mt p53 subgroup. This analysis provides evidence that NF-κB may interact predominantly with p53 in mediating distinct or common pathways in tumor cells with different p53 status.

**Table 2:** Enriched signal pathways in the gene regulatory programs of NF-κB and p53.

HNSCC cell lines	Enriched signaling pathways
mtp53	CDK5, HER-2 in breast cancer, Protein kinase A, Small cell lung cancer
wt p53-deficient	14–3–3-mediated, HIF1α, Leukocyte extravasation, mTOR, NF-κB, TGFβ, VEGF, Xenobiotic metabolism
mt and wt p53	Ephrin receptor, ERK/MAPK, HGF, IGF-1, IL-6, IL-8, Integrin linked kinase, Integrin, Neuregulin, p38 MAPK, p53, PI3K/AKT, PPAR, PTEN, TREM1

Canonical pathways in the table were significantly enriched (*P*<0.05) among the regulatory programs consisting of common target genes between NF-κB and p53.

Finally, we defined two regulatory programs consisting of common target genes of all the seven TFs (Figure 2B). The program of the wt p53-deficient cells comprised 37 genes, where 17 and 12 genes are consistent with known NF-κB and p53 targets, respectively. The percentage of known NF-κB and p53 target genes in the network was greater than their overall prediction (for example, NF-κB: 46% vs. 19%; p53: 32% vs. 15%). Similarly, a set of 39 genes, including 12 known NF-κB and 17 known p53 targets, formed the regulatory programs of the mt p53 cells. The prediction of the known target genes in the program was also relatively accurate by comparing with the overall prediction for NF-κB (31% vs. 15%) and p53 (44% vs. 29%) target genes. In the both transcriptional regulatory programs, 10 target genes were shared between the two subgroups of HNSCC (Figure 2B). Furthermore, we found that most genes in the regulatory programs were functionally classified to adhesion, angiogenesis, apoptosis, cell cycle, inflammatory and immune responses, proteolysis, and regulation of transcription. This indicates that the HNSCC-specific gene programs of the seven TFs can mediate broad functional processes.

### miRNA target genes and their interaction with NF-κB and p53

mir21 and mir34ac are known for their relationships with the p53 or NF-κB pathway. We applied the model-based approach to analyze their target genes and determine how many of these genes are co-targeted by the two TFs using the same gene expression profile of HNSCC cell lines. Table S4 lists the predicted targets of the two miRNAs overlapped with that of NF-κB and p53. We identified 73 and 84 target genes of mir21 and mir34ac in the wt p53-deficient cell lines, respectively. They displayed a highly frequent intersection (56–77%) with target genes for the two TFs. Compared with miRNA databases TarBase and mir2disease, besides the known binding targets, some predicted genes are likely regulated by mir21or mir34ac based on other validation methods such as qRT-PCR, microarray, proteomics and Western blot analyses. For example, *CDKN1A, PIK3R1, RHOB, TGFB1* and *THBS1* are possible targets of mir21; and *EHD1, NDRG1, S100A2*, and *SFN* are likely targets of mir34ac. Moreover, we compared our results with those predicted by software package miRecords, which provides eleven computational methods for miRNA binding target search [37]. Totally 16 and 31 target genes of mir21 and mir34ac in our prediction were identified by at least three methods respectively (Table S4). Similarly in the mt p53 cell lines, the new model predicted 76 and 77 target genes of mir21 and mir34ac, respectively, whereof 63–88% are also targets of the two TFs. By searching miRecords, we also found that 15 targets of mir21 or 29 targets of mir34ac can be detected by at least three computational methods (Table S4).

**Figure 3:**
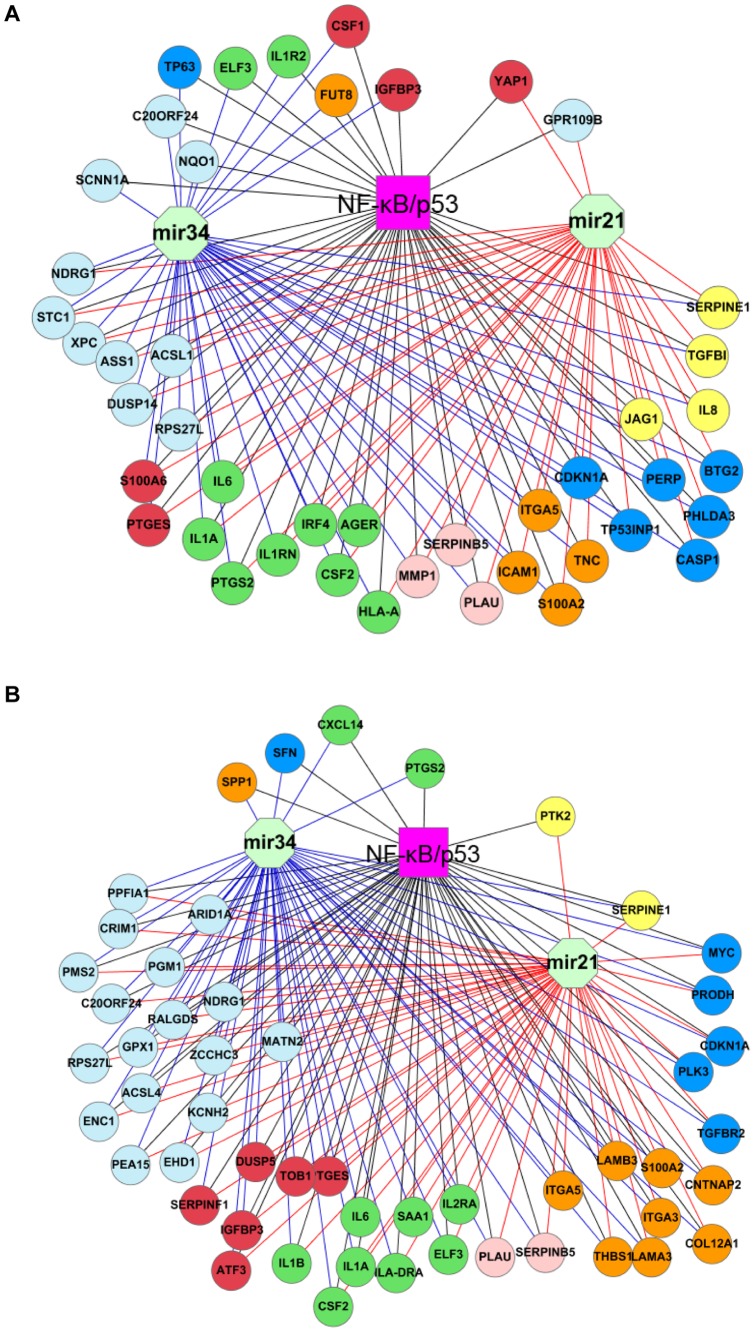
Gene regulatory networks of NF-κB, p53, mir21 and mir34ac in the HNSCC cell lines. A, network of the wt p53-deficient cells. B, network of the mt p53 cells. Every node represents a common target gene of NF-κB, p53, mir21 or mir34ac, and was annotated according to inflammatory and immune responses (green nodes), apoptosis (blue), angiogenesis (yellow), proliferation (red), adhesion (gold), proteolysis (light red) and other processes (light blue). The networks were presented by cytoscape.

We constructed gene networks composed of co-targets of NF-κB, p53, mir21 or mir34ac in the two types of tumor cells. There are 17 common genes presented in the two networks (Figure 3). In the network of the wt p53-deficient (Figure 3A), we identified several known targets of miRNAs, such as *BTG2, JAG1, SERPINB5,* and *TP63* for mir21, and *ACSL1* and *JAG1* for mir34a. The network of the mt p53 includes known targets of mir21 (*SERPINB5* and *TGFBR2*) and mir34ac (*ACSL4* and *MYC*, Figure 3B). This result highlights a considerable interaction of NF-κB and p53 with the two miRNAs for gene regulation in HNSCC cell lines. The majority of the two networks involve in biological functions similar to TF regulatory programs (Figure 2), such as apoptosis, proteolysis, proliferation, cell adhesion and migration, inflammatory and immune responses, and angiogenesis. These cellular processes are possibly related with tumor progression and metastasis [38], [39].

**Figure 4:**
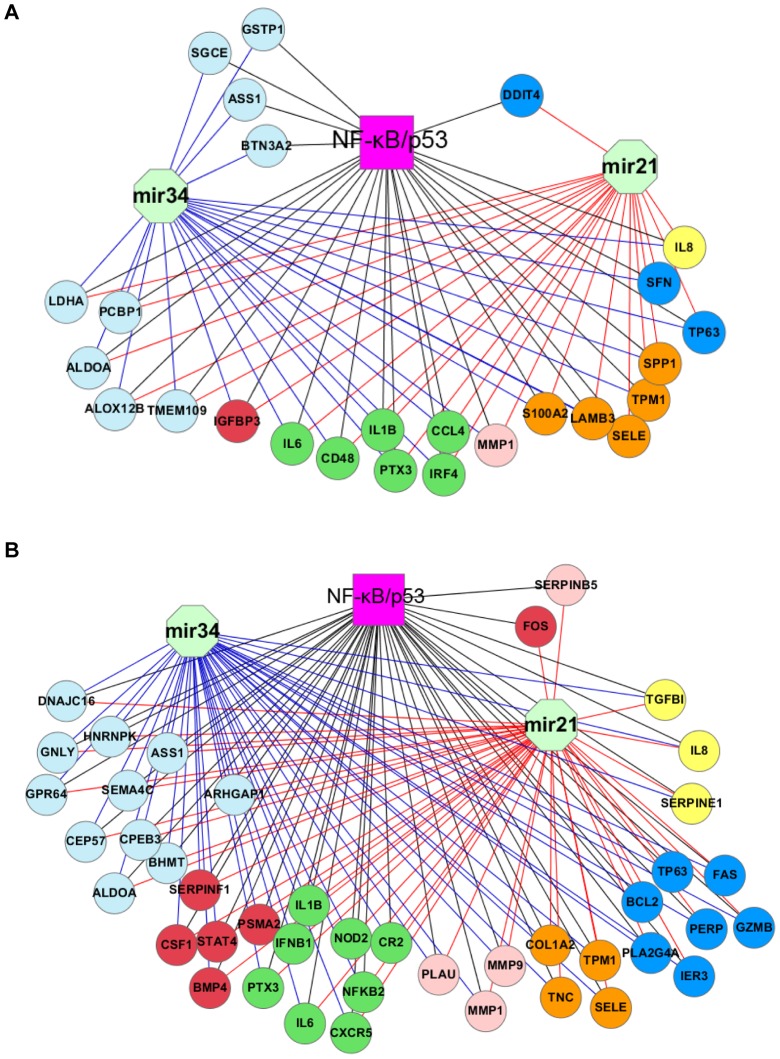
Gene regulatory networks of NF-κB, p53, mir21 and mir34ac in the HNSCC metastatic tissues. A, a network of hypopharyngeal cancer. B, a network of oral cancer. Every node represents a common target gene of NF-κB, p53, mir21 or mir34ac, and was annotated to inflammatory and immune responses (green nodes), apoptosis (blue), angiogenesis (yellow), proliferation (red), adhesion (gold), proteolysis (light red) and other processes (light blue). The networks were presented by cytoscape.

We subsequently sought to construct similar networks of TF-miRNA by analyzing gene expression profiling data from metastatic and non-metastatic tissues in hypopharyngeal [40] and oral cancer [41]. Table S5 lists all overlapped target genes among NF-κB, p53 and the two miRNAs. We constructed two networks consisting of 26 and 43 target genes of the four regulators in the two types of tumor tissues, respectively (Figure 4). By comparison with the cell line networks (Figure 3), 12 and 13 intersected genes in the networks of two types of HNSCC tissues were observed respectively. Also, biological functions in these gene networks are similar, supporting the consistence between cell lines and tissues to some extent. Moreover, we identified about a half of genes which are known targets of NF-κB or p53 in the both networks. For example, *IL6, PTX3, BCL2, FAS, FOS, IER3, PTX3, SERPINE1,* and *TP63* were known to be co-regulated by the two TFs. Interestingly, apoptotic regulator *BCL2* is a known target of both mir21 and mir34ac (Table S5). Our findings indicate that both mir21 and mir34ac likely involve or interact with NF-κB and p53 networks through their downstream genes underlying tumor progression.

### Validation of p53 and NF-κB target genes by siRNA knockdown and promoter binding assay

**Figure 5:**
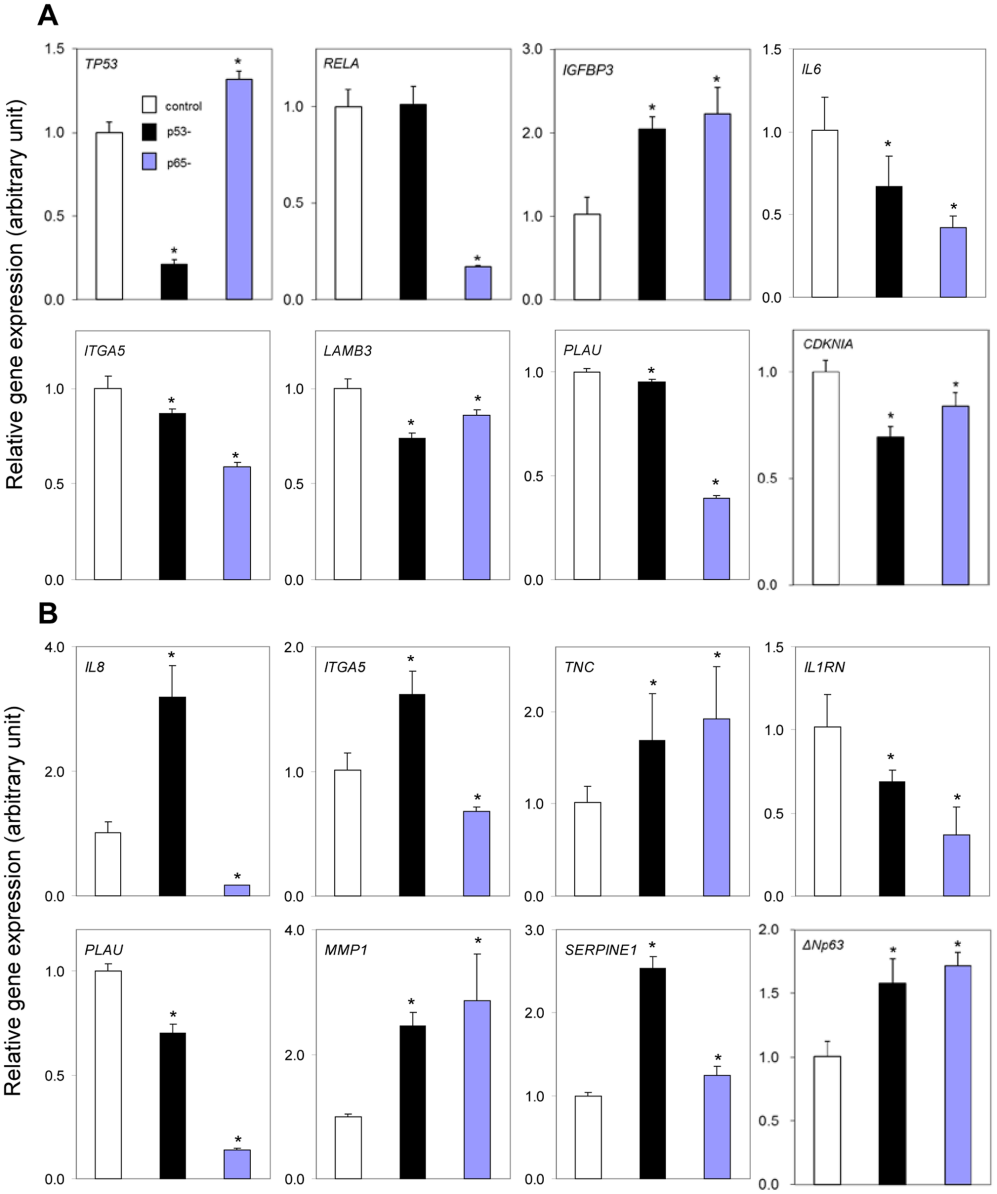
Silencing *RelA*and*TP53* by siRNA significantly altered gene expression. UM-SCC 22B (A, the mt p53) and 1 (B, the wt p53-deficient) cells were transfected with siRNA to *RelA* or *TP53* for 48 h or 72 h. Total RNA was isolated, and genes selected from TF-miRNA networks in the Figure 3 were analyzed by real time qRT-PCR. The data were calculated as the mean plus standard deviation from triplicates with normalization by 18S ribosome RNA, and presented as the comparison with the cultured cells transfected with the control siRNA oligos. The open bar represents the cells transfected with control siRNA, and the values have been normalized to 1. The fold changes of the target genes were presented when compared with those transfected with control siRNA. The black bar represents cells transfected with siRNA targeting TP53, while the blue bar represents cells transfected with siRNA targeting NF-κB subunit, RelA/p65. * refers to statistical significance (t test, *P*<0.05).

We selected the identified selected cancer-related target genes with potentials under p53 and NF-κB regulation in the networks from Figure 3, and tested whether their expression can be affected by siRNA knockdown of either *TP53* or NF-κB *RelA/p65*. As shown in Figure 5A or 5B, there are eight panels each representing eight potential target genes for TP53 or NF-κB from our bioinformatics prediction. The siRNA knockdown experiments were performed in two cell lines, one is UM-SCC 22B with mt p53 (Y220C) but remaining residual p53 binding activity (Figure 5A), and other is UM-SCC 1 with deficient wt p53 status [42] (Figure 5B). The deficient wt p53 status was defined as wild type of p53 genotype but minimal p53 protein expression and binding activity [42]. In each panel, the open bar represents the cells transfected with control siRNA, for which the value has been normalized to 1 to enable comparison. Black bars represent cells transfected with siRNA to knockdown TP53, and the blue bars represent cells transfected with siRNA to knockdown NF-κB subunit RelA/p65. The relative expression after either knockdown of TP53 (black bar) or RelA/p65 is presented as the fold increase or decrease when compared with cells transfected with control siRNA (open bar). In this experiment, previous experimentally validated known target genes of p53 (*CDKN1A* and *IGFBP3*) and NF-κB (*IL6* and *IL8*) are included as positive controls. * indicates statistical significance when compared the gene expression after knockdown of TP53 or RelA/p65 with those from cells transfected with control siRNA. As shown in Figure 5A, after knocking down of *TP53* or *RelA* in UM-SCC 22B cells (mt p53) for 48 hours, the expression level of *TP53* or *RelA* was dramatically reduced by more than 80%, when compared to control siRNA (the upper left two panels). These two panels exhibited the specific and efficient knock down of *TP53* or *RelA/p65* genes. Knocking down *TP53* (black bar) increased the expression of *IGFBP3*, suggesting that p53 mediated suppression of the expression of this gene (upper left third). Silence of *TP53* (black bars) slightly decreased the expression of *IL6, ITGA5, LAMB3, PLAU,* and *CDKN1A* in UM-SCC 22B cells with statistical significance. This could be explained by its mt p53 status with residual function, or as suggested by other findings, that a group of loss of function p53 mutants have gained the ability to interact and promote the transcription of other TFs, including NF-κB, which could promote tumorigenesis [43], [44]. After knocking down *RelA/p65* (Figure 5A, blue bars), most genes were significantly decreased, except *IGFBP3*, which is consistent with NF-κB activation in the cells. Figure 5B showed the knockdown experiments in UM-SCC 1 cells with wt p53 genotype, but where expression of p53 is barely detectable. Further knocking down of *TP53* (black bars) significantly increased the expression of *IL8, ITGA5, TNC, MMP1, SERPINE1,* and *ΔNp63*, but reduced the expression of *IL1RN* and *PLAU*. Our data suggest that the low level of wt p53 protein expression in the cell line is still functional. The data is consistent with previous findings from our and other laboratories, that p53 protein is extremely powerful to regulate gene expression [36], [44]. Knockdown of *RelA* (blue bars) decreased expression of *IL8, ITGA5, IL1RN* and *PLAU* but resulted in an increase in the expression of *TNC, MMP1, SERPINE1* and *ΔNp63* (Figure 5B), which are consistent with our predictions as NF-κB target genes.

**Figure 6:**
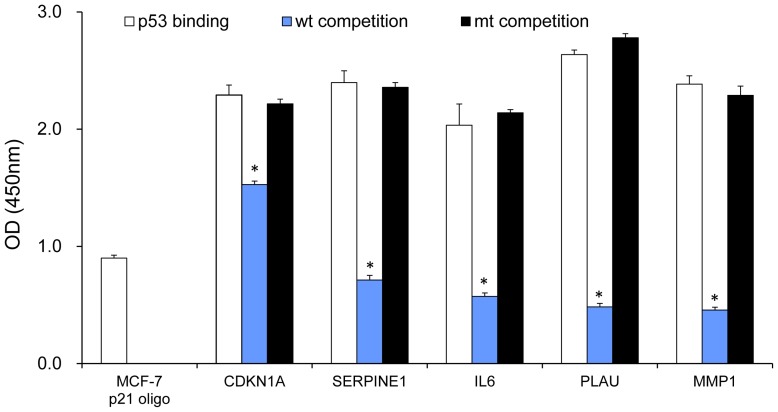
Binding activity of p53 on the promoters of selected genes. Nuclear extract from UM-SCC 1 cells were isolated, and the binding activity was examined on the biotin labeled oligos containing promoter sequences with p53 binding sites. The binding activity of nuclear extract of MCF-7 cells with a biotinylated oligonucleotide containing a known p53 binding site in the *CDKN1A* gene promoter (“MCF-7 p21 oligo”) was served as a positive control. Specificity of binding in the UM-SCC cells was also tested using cold competition (unbiotinylated) oligonucleotides which contained the wild-type p53 consensus binding sequence (“wt competition”) or mutant DNA sequence of p53 binding site (“mt competition”).The mean and standard deviation of each binding was calculated from triplicate, and the presented data are from one representative of repeated experiments. *refers to statistical significance when p53 binding activity was competed by the mt oligo, to confirm the binding specificity (t test, *P*<0.05).

Previously, we analyzed binding activity of NF-κB subunits RelA and NFκB1 in three UM-SCC cell lines using TransAM NF-κB oligonucleotide-ELISA binding assay from Active Motif [16]. Here we examined binding activity of p53 on the promoters of its target genes identified in this study, using the newly modified p53 oligonucleotide-ELISA binding assay, based on TransAM p53 ELISA from Active Motif. In this modified assay, p53 binding consensus sequence was replaced by different promoter sequences with predicted p53 binding sites (detailed information in Methods). We used MCF-7 cell lysate as the positive control, which was provided by Active Motif. We used an oligo containing a known p53 binding site in the *CDKN1A* promoter (“MCF-7 p21 oligo”, in Figure 6) for a positive control [45]. This oligonucleotide contains a 63-bp sequence containing known p53 binding motif, which has been validated previously by Electrophoretic Mobility Shift Assay (EMSA) and ChIP binding assay result of *CDKN1A/p21* promoter [20]. Since we previously showed that UM-SCC 1 (wt p53 deficient) and UM-SCC 22B (mt p53) exhibited minimal p53 binding activity [42], we overexpressed wt p53 in UM-SCC 1 cells to test p53 binding activity for the predicted p53 binding motifs on the promoters of less studied p53 targeted genes using Genomatix MatInspector. We found strong p53 binding activities on predicted p53 sites on *CDKN1A, SERPINE1, IL6, PLAU* and *MMP1* promoters (Figure 6). Using excess p53 wild type (wt) oligo in the binding assay, we were able to compete off the binding activity, while the mutant (mt) oligo did not compete off the binding activity, demonstrating the binding specificity (Figure 6). Our experimental data confirmed the predicted binding motifs of the new p53 target gene.

## Discussion

Our method is regulatory component analysis model-based and can capture the sparse structure existing in genomic-scale gene expression data for unraveling gene regulatory networks. The advantage of the newly developed method is supported by the improved prediction of NF-κB and p53 target genes, in comparison with other methods. First, we compared our method to a Bayesian model-based COGRIM. Among the NF-κB targets predicted by our method, of 19% (in the wt p53-deficient) and 15% (in the mt p53) are consistent with known ones published previously. By contrast, the known NF-κB genes predicted by COGRIM only reached to 10% of the total prediction [16]. Importantly, the NF-κB genes predicted by our method are more functionally relevant than those by COGRIM (Table 1). Second, we analyzed the percentage of known p53 target genes in its total prediction. Previous studies have shown that p53 binding could be one of key elements controlling over-expression of genes in the mt p53 cells of HNSCC [15], [34]. Our analysis indicates that 10% (in the wt p53-deficient) and 24% (in the mt p53) of the over-expressed p53 target genes are matched with known ones, thus partially supporting the previous finding. Some high-throughput technologies, such as ChIP-pet [46] and ChIP-seq [47] were utilized to detect physical binding sites of p53 on whole genomic sequences. Wei et al. identified 542 p53 binding loci clustered in 458 known genes by using p53-ChIP DNA fragments and overlapping pet-clusters as the readout [46]. By searching for known p53 target genes, we found that 15% (in the wt p53-deficient) and 29% (in the mt p53) of the total p53 targets predicted by our method are identical, which is relatively better than the ChIP-pet method (14%). Recently, a genome-wide analysis of p53 binding sites in normal human IMR90 fibroblast cells showed 743 high-confidence ChIP-seq peaks representing 956 genes [47]. We compared these putative genes with known p53 targets, and found 52 overlapped ones, which is less than our predication. Taken together, our method improves the efficiency and accuracy of identification of regulatory associations between NF-κB or p53 and their target genes. The identified NF-κB genes by the newly developed method are highly associated with biological processes altered in HNSCC, suggesting that they are biologically more meaningful than those by other methods.

Two master TFs, NF-κB and p53, play different roles in human cancer, in which activation of p53 and inhibition of NF-κB promote apoptosis. Their crosstalk is suggested as an important mechanism for regulating molecular oncogenesis [11]. However, how they interact and globally modulate gene networks remains unclear. In the present study, we demonstrate a significant intersection between p53 and NF-κB regulated genes in the HNSCC cell lines. Such interaction is different in the two tumor subgroups. In the wt p53-deficient cells, the two TFs can jointly regulate over 60% of their targets in the under-expressed genes (Figure 2A). The two factors, along with the other five TFs (AP1, CEBPB, EGR1, SP1 and STAT3), are implicated in modulating 90% of the under-expressed NF-κB target genes (Table S1). However, in the mt p53 cells, all of the predicted p53 targets can be regulated by NF-κB (Figure 2A). Significantly, the three subunits RelA, NFκB1 and cRel cooperate with p53 to regulate their common genes, which facilitates a wide interplay between p53 and NF-κB networks. This observation strongly suggests that a tight interaction between NF-κB family and p53 controls p53 networks in the mt p53 tumor cells, but to a lesser extent in the wt p53-defieient cells. On the other hand, most (about 80%) of the predicted NF-κB target genes in the mt p53 cells are not regulated by p53 (Figure 2A). A half of these NF-κB genes are also not targeted by any one of other five TFs (Table S1). Therefore, the NF-κB genes in the mt p53 tumor cells are possibly modulated by loss or altered of p53 function, or by means of interactions of NF-κB with a broad arrange of TFs or miRNAs.

We identified two transcriptional gene regulatory programs that are likely modulated by the seven TFs (Figure 2B). Previous experimental studies on TF interactions in gene regulation support our discovery, for example, NF-κB-AP1-CEBPB [22], [27], NF-κB-STAT3 [26], NF-κB-AP1-EGR1 [29], NF-κB-AP1-SP1 [30], NF-κB-AP1-p53 [48], etc. In HNSCC cell lines, co-binding of RelA, AP1 and CEBPB on *IL8* promoter contributed to its over-expression [22]. A similar result was repeated in breast cancer cells [27], indicating that *IL8* up-regulation requires a complex modulation among the three TFs. Apart from *IL8*, the co-regulation between NF-κB and AP1, CEBPB or STAT3 for genes *IL1B*, *ICAM1, IL6, PTGS2*, and *SAA1* in the TF regulatory networks (Figure 2B) is consistent with previous observation in HNSCC or other cancer cells [49], [50], [51], [52], [53], [54]. By promoter analysis, the co-binding motifs of NF-κB with AP1, STAT3 and EGR1 has been identified on clustered genes over-expressed in the HNSCC cell lines [15]. In addition, we observed that a larger number of predicted NF-κB and p53 target genes in the two inferred gene regulatory programs are consistent with the known ones (Figure 2B), by comparison with the overall prediction of their target genes. This result provides evidence for the accuracy of inferred regulatory programs in the HNSCC cell lines. Through experimental examination on the targets of NF-κB and p53 involved in these networks, we confirm that their expression was significantly altered in the both wt and mt p53 tumor cell lines after the knockdown of *RelA* and *TP53* (Figure 5). Thus, we experimentally validated a group of newly predicted NF-κB or p53 target genes, which support the prediction power of this new system biology approach to integrating multiple platforms and data involved in the transcriptional and regulatory interactions among NF-κB, p53 and the other TFs.

Our results demonstrate that the target genes in the transcriptional regulatory programs mediate a variety of functional processes, consistent with previous experimental reports on NF-κB genes that regulate apoptosis, adhesion, angiogenesis, inflammatory and immune responses, proliferation, and migration in HNSCC cells [15], [16], [22], [55].The co-target genes of NF-κB and p53, such as *CAKN1A, IGFBP3, IL1A, IL1B, IL1RN, IL6, IL8, ITGA3, ITGA5, LAM3A, LAM3B, PTGS2, SFN*, are presented significantly in enriched pathways in the Table 2 have been examined experimentally in this study (Figures 5, 6), and from previous reports [16], [56], [57], [58]. Distinct signal pathways identified are more specific to different phenotypes in the tumor cells related with different p53 status (Table 2). For example, genes significantly enriched in TGFβ pathway are repressed in the tumor cells with the wt p53-deficient status (Table S3B). This finding is consistent with the negative regulatory function of TGFβ in HNSCC cells, observed by Lu, et al. [59]. Our work establishes a strong link with regulatory programs of NF-κB and p53, and their related pathways or functional processes, in agreement with many conclusions previously drawn from the biologic experiments.

Discovery of TF and miRNA interactive modules can advance our understanding of complex transcriptional regulatory architectures in cancer cells. We chose mir21 and mir34ac in light of their involvement in p53 or NF-κB signaling pathway for reconstructing transcriptional and post-transcriptional gene regulatory networks. The over-expression of oncogenic mir21 was constantly observed in 15 of 16 microarray studies based on diverse HNSCC samples whereas aberrant expression of tumor suppresser mir34 family was identified in 6 of these studies (data not shown). It is reasoned that the two miRNAs are likely involved in transcriptional modulation of NF-κB or p53 during HNSCC development. We constructed two gene networks regulated by the four factors in the cell lines of HNSCC (Figure 3). To assess our result, we first identified a large fraction of network genes which are known NF-κB target genes. These genes mediate immune and inflammatory responses (*CSF2, IL1A, IL1B, IL1RN, IL1R2, IL6*, and *IL8*), apoptosis (*CDKN1A* and *IGFBP3*), and adhesion (*ITGA5, LAMA3, LAMB3* and *ICAM1*), validated in HNSCC cell lines (Figure 5, and [16]). Moreover, some of known p53 binding targets such as *CDKN1A, IGFBP3, PERP, PTGS2, S100A2, SERPINB5, SERPINE1, SFN*, and *TP63* are also components of the networks. Second, we have experimentally confirmed a number of predicted targets of p53 in the networks by silencing of *TP53* and testing binding activities of p53 on their promoters in the tumor cells (Figures 5, 6). p53 binding activity assay on the gene promoters provides functional evidence for the regulatory networks (Figure 6). This newly developed assay mimics traditional gel shift assay (EMSA), using DNA oligos with biotin labeling instead radioisotope (Active Motif). The advantage of this assay over commonly used ChIP assay is using more defined binding oligo sequence that contains only 40–60 nucleotides. However, in ChIP assay, the DNA sequence involved in the TF and antibody complex is usually 100–500 bp long, where many potential binding activities may be involved with other binding motifs [60]. Third, it is noticeable that more than a half of genes in the TF regulatory programs (Figure 2B) overlap with the networks of TF-miRNA (Figure 3), demonstrating a coordinated interaction of mir21 and mir34ac with multiple TFs contributing to transcriptional regulation of genes. Several known or likely target genes of mir21 or mir34ac were identified in the networks, providing evidence for the miRNA involvement. Two genes *SERPINB5* and *TP63* are known targets of mir21 and p53, where *TP63* is also a transcriptional target of NF-κB [61]. Thus, *TP63* is likely a key gene interconnected by NF-κB, p53 and mir21. On the other hand, as one of the p53 family members, the TAp63 isoform is able to exert biological functions similar to those of p53 and contributes to oncogenesis and tumor-suppressor activity [62]. We have shown that some p53 genes in the networks are also known to be regulated by p63, such as *CDKN1A, IGFBP3, PERP, S100A2, SERPINB5,* and *SFN*. Another example gene *JAG1* is a known binding target of mir21, mir34a and p63, supporting the potential of p63 interacting with TFs and miRNAs in gene regulation of HNSCC. Recently, we demonstrated that ΔNp63 interacts with NF-κB subunit cRel or RelA to modulate gene programs mediating apoptosis, cell growth arrest, inflammation, epidermal hyperplasia and the malignant phenotypes of HNSCC cells [20], [36].

Furthermore, we found that many genes in the TF-miRNA networks are related to biological processes contributing to the progression of HNSCC. Several common target genes in the networks were down-regulated in both wt and mt tumor cells (Figure 3, Table S4), such as *ITGA5* and *S100A2* (adhesion and migration), *SERPINE1* (angiogenesis), *CDKN1A* (growth arrest), and *PLAU* and *SERPINE5* (proteolysis). These cellular processes possibly favor HNSCC metastasis [39]. The two miRNAs may interact with p53 or NF-κB to modulate their target expression so that repressing progression of HNSCC. By contrast, the over-expressed genes in the networks may trigger tumorigenesis of HNSCC by altering gene expression associated with inflammatory, proliferation, apoptosis and other processes (Figure 3, Table S4), such as *IL6, IGFBP3, ELF3*, and *PTGES* in the both subgroups of tumor cells. The miRNAs and TFs likely mediate metastatic processes through the increased expression of their target genes, such as *IL8, ICAM1* and *TGFBI* in the wt p53-decifient cells, or *SPP1* and *PTK2* in the mt p53 cells (Figure 3, Table S4). To validate the results in the cell lines, we constructed two regulatory networks by analyzing datasets of metastatic and non-metastatic HNSCC tissues (Figure 4). Several important genes associated with tumor metastatic program, such as *IL6, IL8, MMP1, PLAU, SERPINB5, SERPINE1, SPP1, TGFBI, TNC* and *TP63* were consistently confirmed in the both tumor cell lines and tissues.

miRNAs are implicated in tumor progression, as metastasis activators or suppressors [63], [64]. mir21, as a master regulator of the metastatic processes was found to stimulate cell invasion by targeting tumor suppressors *TPM1, PDCD4* and *SERPINB5* in breast cancer [65] or colon cancer [66]. As the mediator of tumor suppression by p53, mir34 family may contribute to the inhibition of invasion or metastasis in various cancer types [67]. mir34ac could serve as a metastasis suppressor to regulate breast cancer migration and invasion through targeting oncogene Fos-related antigen 1 [68]. In human malignant melanoma, an over-expression of mir34ac suppresses invasive growth of tumor cells with wild-type p53 gene [69]. In the networks of HNSCC metastatic tissues (Figure 4), we identified several representative miRNA targets *BCL2, MMP9, SERPINE5, TPM1*, and *TP63*, consistent with the previous experimental results derived from miRNA target databases TarBase and mir2disease. The anti-apoptotic *BCL2*, a known binding target of mir21 and mir34ac, is co-regulated by NF-κB and p53 in oral metastatic tissues (Figure 4B), suggesting a model for the interaction between TFs and miRNAs in facilitating tumor progression and metastasis. Taken together, our analyses provide a view of cross-regulatory relationships among NF-κB, p53 and the miRNAs in different malignant phenotypes. To our knowledge, this is the first investigation of TF-miRNA regulatory interactions by modeling diverse data sources in HNSCC. Within experimental validation of predicted miRNA targets, it should help understanding of transcriptional and post-transcriptional regulatory mechanisms responsible for heterogeneity of HNSCC.

## Conclusions

In summary, our analysis indicates that two master TFs, NF-κB and p53, have a wide impact on distinct or shared biological functions in HNSCC cells, through a coordinated interaction to regulate gene expression programs. A certain number of the identified genes have previously been examined as known NF-κB or p53 targets, or validated by siRNA knock down of the two TF genes and p53 binding activity assay. Furthermore, we have found that NF-κB, p53 and two miRNAs, mir21 and mir34ac, may constitute concerted regulatory modules and play an important role in modulating downstream gene networks contributing to metastatic processes of HNSCC. Through the integrated analyses, our studies provide a framework for experimental analysis of TF-miRNA regulatory modules, and insights into the complex regulatory mechanisms underlying squamous cell carcinoma. The identified gene programs are highly associated with cancer-related pathways and functions, suggesting that the newly developed method is suitable to inferring the gene regulatory networks in these cancer data. Application of the new methodology in HNSCC has showed that it can be utilized as a useful approach to study on other biological complex systems.

## Supporting Information

Figure S1 **A map of transcriptional regulatory network in HNSCC cell lines.** In the figure, every node (grey one) represents a target gene of at least one of TFs RelA/p65, NFκB1/p50, cRel, p53, AP1, CEBPB, EGR1, SP1 and STAT3 (pink nodes). A, the wt p53-deficient cells. B, the mt p53 cells.
(PDF)

Table S1 **List of TF target genes predicted by regulatory component analysis model-based method.** The genes in red and green represent differentially over- and under-expressed with fold change ≥2.0 in HNSCC cell lines of the wt or mt p53.
(PDF)

Table S2 **Overlapping of target genes between NF-κB or p53 and other TFs in HNSCC cell lines.**
(PDF)

Table S3 **Significant enrichment of biological functions among common target genes between NF-κB and p53 in HNSCC cell lines.** Joint target genes of NF-κB and p53 were annotated to, A. GO biological processes by using DAVID tool with P<0.001, FDR <2%. B, Canonical signaling pathways by using Ingenuity Pathway Analysis tools (P<0.05). Genes in red and green indicate the over- and under-expressed genes in HNSCC cell lines above at least 2 fold changes, respectively.
(PDF)

Table S4 **Target genes of mir21 and mir34ac and their overlapping with NF-κB and p53 targets in HNSCC cell lines.** Based on miRNA databases TarBase and mir2disease, “target validated” refers to target genes of mir21 or mir34ac tested by reporter assay, and “target likely” refers to genes of mir21 or mir34ac tested by other methods (such as microarray, qRT-PCR, Western blot, and proteomics). The number in the parenthesis refers to number of computational methods (≥3) for the miRNA target gene prediction based on miRecords. The genes in red and green represent differentially over- and under-expressed at least fold change 2.0 in the wt and mt p53 cells.
(PDF)

Table S5 **Overlapped target genes of NF-κB, p53, mir21 and mir34ac in HNSCC tissues. (Legends see Table S4).**
(PDF)

Text S1 **Binding data of TFs and miRNAs.**
(PDF)
