# Supplementary figures and images for: Unraveling Regulatory Programs for NF-kappaB, p53 and MicroRNAs in Head and Neck Squamous Cell Carcinoma
Source: PLoS One. 2013 Sep 19;8(9):e73656. doi: 10.1371/journal.pone.0073656 (PMC3777940; doi:10.1371/journal.pone.0073656)

A

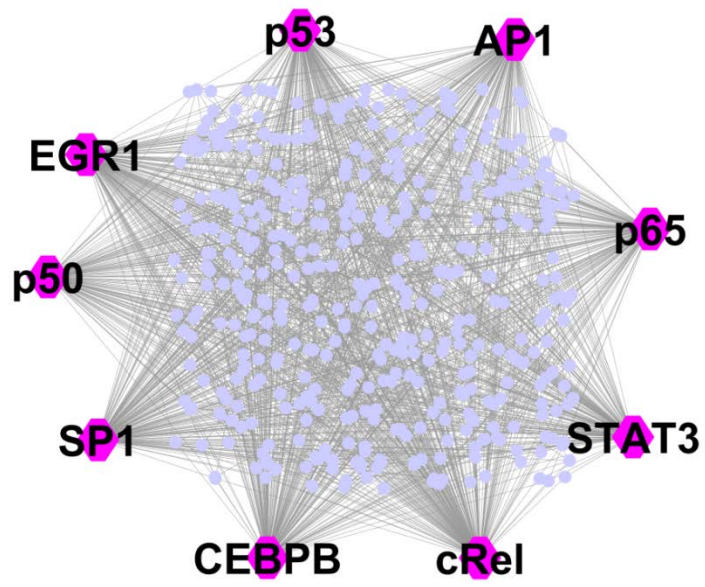

B

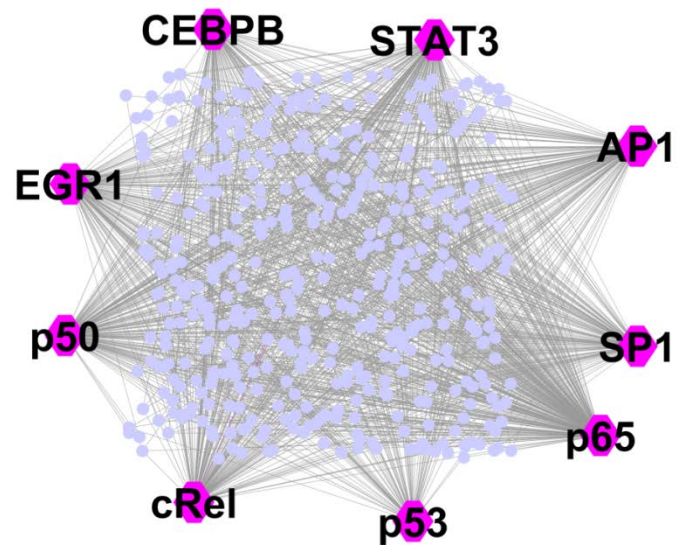

Supplement: Figure S1 — A map of transcriptional regulatory network in HNSCC cell lines. In the figure, every node (grey one) represents a target gene of at least one of TFs RelA/p65, NFκB1/p50, cRel, p53, AP1, CEBPB, EGR1, SP1 and STAT3 (pink nodes). A, the wt p53-deficient cells. B, the mt p53 cells. (PDF) [file pone.0073656.s001.pdf]
