# Supplementary material for: Unraveling Regulatory Programs for NF-kappaB, p53 and MicroRNAs in Head and Neck Squamous Cell Carcinoma
Source: PLoS One. 2013 Sep 19;8(9):e73656. doi: 10.1371/journal.pone.0073656 (PMC3777940; doi:10.1371/journal.pone.0073656)
Supplement: Table S1 — List of TF target genes predicted by regulatory component analysis model-based method. The genes in red and green represent differentially over- and under-expressed with fold change ≥2.0 in HNSCC cell lines of the wt or mt p53. (PDF) [file pone.0073656.s002.pdf]

Differentailly over-expressed genes in wt p53-deficient HNSCC cell lines

Gene symbol	RelA	cRel	NFκB1	p53	CEBPB	STAT3	AP1	EGR1	SP1
AARS				taget				taget	
ABCG2		taget				taget			
ACSL5	taget	taget	taget		taget	taget		taget	taget
ADA							taget		
AKAP12	taget	taget		taget	taget	taget	taget	taget	taget
ALB	taget	taget	taget		taget	taget		taget	taget
ALDH1A3	taget	taget	taget	taget		taget		taget	
ANXA9	taget		taget						
APIP							taget		taget
APOL1			taget						
APOL3			taget						
ARHGDIB					taget				
ARID3A							taget		
ARL6IP1								taget	
ATAD2	taget	taget	taget		taget	taget		taget	
ATP9A				taget					
AURKA					taget	taget			
AZIN1					taget	taget	taget		taget
BCAN	taget		taget						
BIRC2		taget	taget			taget	taget	taget	
BLMH							taget		
C1ORF61	taget								
C1QTNF6			taget	taget					
C1S		taget	taget	taget	taget	taget	taget		taget
C20ORF11									taget
C20ORF199			taget	taget					
C20ORF24		taget	taget		taget		taget		
C7ORF30								taget	taget
CA9		taget							
CAP2							taget		
CASP1	taget	taget	taget	taget	taget	taget	taget	taget	taget
CASP4		taget		taget			taget		
CBX3						taget			taget
CCNB2						taget			
CD151	taget	taget	taget						
CDC20		taget			taget	taget			
CDC45L							taget		
CEACAM7				taget					
CENPA							taget		
CENPN							taget		
CENTD3							taget		
CEP110				taget					taget
CGN	taget						taget		
CHCHD2				taget				taget	taget
CHKA	taget	taget		taget	taget	taget	taget	taget	taget
CIT	taget		taget				taget		
CLDN3		taget			taget	taget			
CLDN4		taget		taget		taget			
CLDN7		taget	taget						
CNAP1	taget				taget				
CP	taget	taget	taget	taget	taget	taget		taget	taget
CPSF5							taget		taget
CPVL		taget							
CRIP2			taget				taget		
CSF1	taget	taget	taget	taget	taget	taget			

CTSA			taget	taget					
CTSH				taget				taget	
CYB561							taget		
CYBA	taget		taget						
DBNDD2				taget			taget		taget
DDEF1		taget			taget	taget			
DDX47	taget		taget	taget	taget	taget	taget	taget	taget
DHODH							taget		
DHRS3				taget				taget	
DHX35									taget
DIDO1	taget								
DIO2	taget	taget	taget	taget					
DMAP1				taget		taget			
DNAH11								taget	
DPH2L2				taget					
DPP7	taget			taget					
DUSP10		taget				taget			
EHMT2							taget		
EIF4G1	taget			taget	taget	taget	taget	taget	
ELF3	taget	taget	taget	taget	taget	taget	taget	taget	taget
EPHB2				taget		taget		taget	taget
EPPB9								taget	
EPS8	taget								
ERGIC3				taget			taget		
ETV1	taget	taget	taget	taget	taget	taget	taget	taget	taget
F8A1							taget		
FADS3				taget				taget	
FAM115A		taget		taget		taget			
FAM60A									taget
FARP1							taget		
FBXL11	taget	taget	taget	taget	taget	taget	taget	taget	taget
FBXL14	taget	taget	taget		taget	taget	taget	taget	taget
FBXO5							taget		
FOXM1	taget				taget				
FUT8		taget	taget		taget	taget	taget	taget	taget
FYB		taget							
GAA			taget						
GABARAPL1				taget	taget	taget			
GAS8							taget		
GATA2			taget						
GDA			taget						
GINS2							taget		
GLE1	taget				taget		taget		taget
GLIPR1		taget	taget						
GOLPH3				taget	taget	taget			taget
GPR107		taget		taget	taget	taget			taget
GPR109B		taget	taget	taget	taget				taget
GPRC5A		taget			taget	taget			
GPX2		taget	taget	taget				taget	
GULP1		taget		taget				taget	
H19		taget	taget						
HIBADH				taget				taget	
HIST1H4B							taget		
HIST1H4C	taget	taget							
HIST1H4E							taget		
HLA-DOA				taget					
HLTF	taget								
HOXB7	taget	taget	taget	taget	taget	taget	taget	taget	taget
HOXB9	taget	taget		taget	taget	taget	taget	taget	

HOXD10	taget		taget		taget		taget		taget
HTATIP	taget	taget	taget	taget	taget	taget	taget	taget	taget
HTATIP2				taget				taget	
ICAM1	taget	taget	taget	taget	taget	taget	taget	taget	taget
IGF2		taget			taget	taget	taget	taget	taget
IGF2BP1	taget	taget	taget	taget	taget	taget	taget	taget	taget
IGFBP3	taget	taget	taget	taget	taget	taget	taget	taget	taget
IGFBP6	taget				taget			taget	taget
IKBKE	taget	taget	taget					taget	
IL22RA1	taget		taget						
IL32		taget							
IL6	taget	taget	taget	taget	taget	taget	taget	taget	taget
IL8	taget	taget	taget	taget	taget	taget	taget	taget	taget
IVD			taget						
JRKL			taget						
KCNJ8				taget					
KCNN4						taget		taget	
KEAP1							taget		
KHK		taget							
KIAA1648							taget		
KIF23						taget			taget
KIF2C			taget			taget			
KRT18		taget		taget	taget	taget	taget	taget	taget
KRT19		taget			taget	taget			
KRT8			taget						
KRTAP2-4							taget		
LAMP3		taget	taget		taget			taget	taget
LCN2	taget		taget		taget			taget	
LIMCH1	taget		taget						
LMO7	taget		taget						
LRP5			taget						
MAGEA2B		taget							
MAGEB2		taget	taget					taget	
MCCC1	taget		taget						
MCM2							taget		
MGST1		taget			taget	taget			
MRE11A							taget		
MRPS30					taget	taget			taget
MSX1		taget							
MTMR2			taget				taget		
MUC4		taget			taget	taget	taget		
MYBL2	taget				taget				
MYO5B				taget					
NDUFAF1	taget						taget		
NDUFB9		taget				taget			
NECAB3							taget		
NEK2	taget								
NNMT	taget								
NQO1	taget	taget	taget	taget	taget	taget	taget	taget	taget
NUSAP1							taget		
ORC1L							taget		
PALM								taget	
PARP3		taget			taget	taget			
PCCB							taget		
PEX5		taget			taget	taget			
PHF20							taget		
PIM1	taget		taget		taget	taget	taget		taget
PLAT		taget	taget	taget	taget		taget		taget
PLK1						taget			

POLE3							taget		
PPAP2C								taget	
PPARG			taget		taget	taget			
PPP1R12A		taget							
PRIM1		taget							
PSMB10	taget		taget						
PTGES	taget	taget		taget	taget		taget	taget	taget
PTPRA						taget			
PTPRJ		taget			taget	taget	taget	taget	taget
RAB17							taget		
RAD1				taget					
RAD54L									taget
RAE1									taget
RARRES3	taget	taget	taget						
RBBP4		taget		taget	taget	taget	taget	taget	taget
RECQL4							taget		
RFC4							taget		taget
RNF139				taget		taget			taget
RPA3	taget	taget	taget		taget	taget		taget	
RPN2		taget				taget		taget	
RPS4Y1	taget	taget			taget	taget			
SAPS3	taget		taget				taget		
SCCPDH			taget						
SCNN1A	taget	taget	taget	taget	taget		taget	taget	
SCYL1		taget		taget	taget	taget	taget	taget	taget
SEMA4D		taget							
SERPINB1			taget						
SEZ6L2		taget			taget	taget	taget	taget	taget
SHANK2		taget							
SIM2	taget				taget				
SLC12A7				taget					
SLC2A4RG	taget						taget		
SMC2									taget
SNCG	taget							taget	
SRPRB				taget					
SRPX2	taget		taget		taget	taget			
STC1		taget	taget		taget	taget	taget	taget	
STC2			taget		taget	taget			
STK18				taget				taget	
STRAP				taget					taget
SULT1A4							taget		
SUV420H1		taget					taget		
SYT17				taget			taget		
TACSTD1	taget	taget				taget			
TAF7							taget		taget
TAL1	taget	taget	taget	taget	taget	taget	taget	taget	taget
TAX1BP1				taget					taget
TBCD				taget					taget
TBX5		taget		taget	taget	taget	taget	taget	taget
TEAD4						taget			taget
TFRC	taget		taget	taget					
TGDS		taget				taget			
TGFBI						taget	taget		taget
TIMM10			taget				taget		
TK1	taget						taget		
TMEM123		taget				taget			
TNFAIP2	taget	taget	taget		taget				taget
TNP1	taget								
TOMM34					taget	taget			taget

TPX2					taget	taget			
TRAPPC2L							taget		
TROAP							taget		taget
TSC22D1				taget	taget				taget
TSPAN13				taget				taget	taget
TXNRD1						taget			
UBE2C		taget				taget			
UBE2D4			taget						
UBE2L6		taget	taget						
USP13							taget		
VGLL1		taget			taget	taget			
YAP1		taget				taget	taget	taget	
ZCWCC1			taget						
ZNF239	taget								

Differentailly underexpressed genes in wt p53-deficient HNSCC cell lines

Gene symbol	RelA	cRel	NFκB1	p53	CEBPB	STAT3	AP1	EGR1	SP1
ACLY		taget		taget	taget			taget	taget
ADAM8								taget	taget
AGER	taget	taget	taget	taget	taget	taget	taget	taget	taget
AHNAK	taget	taget		taget	taget	taget		taget	taget
ANGPT1		taget		taget	taget			taget	taget
ANXA3				taget					taget
ANXA8L1				taget	taget				taget
APP		taget			taget			taget	taget
AREG								taget	taget
ARSB				taget	taget				
ASS1	taget	taget				taget	taget	taget	
ATP10D									taget
BCL11B						taget		taget	taget
BMP4	taget	taget	taget	taget	taget	taget	taget		taget
BNC1	taget				taget	taget		taget	taget
BTG2	taget	taget	taget	taget	taget	taget	taget	taget	taget
C20ORF10								taget	
CALU	taget	taget	taget	taget	taget	taget	taget	taget	taget
CARD6	taget	taget	taget				taget		
CAV1				taget					
CDK5R1					taget	taget		taget	taget
CDKN1A	taget	taget	taget	taget	taget	taget	taget	taget	taget
CKAP4		taget		taget	taget			taget	taget
CNTN1				taget					taget
COL12A1				taget	taget	taget		taget	taget
COL17A1	taget		taget			taget	taget		taget
CSF2	taget	taget	taget	taget	taget	taget	taget	taget	taget
CSTA	taget		taget		taget	taget			
DGKA						taget		taget	taget
DISC1		taget							
DKK3									taget
DNAJB5		taget			taget			taget	taget
DSE				taget					
DSG3								taget	taget
DUSP14				taget	taget			taget	taget
EFNB1								taget	
EGLN1	taget						taget		
EMB						taget			
EMP1				taget		taget			
ENTPD3	taget	taget							
EPB41						taget		taget	taget
ERCC1									taget

ETHE1						taget			
FBN2	taget	taget	taget	taget	taget	taget		taget	taget
FBXW7		taget		taget	taget			taget	taget
FEZ1								taget	taget
FGFBP1	taget	taget		taget	taget	taget	taget	taget	
FHL1								taget	taget
FST				taget	taget			taget	taget
FYN								taget	
GAP43						taget		taget	taget
GJA1	taget	taget	taget	taget		taget	taget	taget	taget
GJB2	taget		taget	taget	taget		taget		
GJB3					taget				
GJB5	taget	taget			taget				taget
GZF1		taget							taget
HCN4	taget	taget	taget	taget	taget	taget		taget	taget
HLA-A	taget	taget	taget	taget	taget	taget	taget	taget	taget
HR	taget	taget			taget	taget		taget	taget
IFI27	taget					taget	taget		
IFI6						taget			
IL1A	taget	taget	taget	taget	taget		taget	taget	taget
IL1R2	taget	taget	taget	taget	taget	taget	taget		taget
IL1RN	taget	taget	taget	taget	taget	taget	taget	taget	taget
INHBA		taget		taget	taget				
IRF4	taget	taget	taget	taget		taget	taget	taget	taget
ISG15	taget								
ITGA2					taget				
ITGA3	taget	taget		taget	taget	taget	taget		taget
ITGA5	taget	taget	taget	taget	taget	taget	taget	taget	
ITGA6				taget		taget			taget
ITGB4				taget	taget				
IVL	taget	taget	taget	taget	taget		taget		taget
JUND				taget					taget
JUP				taget	taget				
KCND2	taget	taget		taget	taget	taget		taget	taget
KIT				taget		taget		taget	taget
KLK10				taget		taget			
KLK5		taget			taget				taget
KRT14	taget		taget				taget		
KRT16	taget	taget	taget	taget	taget	taget	taget	taget	taget
KRT5	taget		taget						
KRT6A	taget						taget		
KRT6B	taget								
LAD1				taget	taget				
LAMA3		taget	taget	taget			taget		taget
LAMB3	taget		taget	taget	taget	taget	taget		taget
LAMC2	taget		taget		taget		taget		taget
LIMA1									taget
LIPG						taget		taget	taget
LOC100128809								taget	taget
MAFK								taget	
MFAP5			taget					taget	taget
MGLL		taget		taget	taget			taget	taget
MLL	taget			taget	taget	taget		taget	taget
MMP1	taget	taget	taget	taget	taget		taget	taget	taget
MN1	taget	taget		taget	taget	taget		taget	taget
MRC2			taget	taget	taget	taget		taget	taget
MRPL12				taget		taget			
MST1R					taget			taget	
MT1L				taget	taget				

MT2A				taget	taget				
MX1	taget						taget		
NDRG1		taget	taget	taget	taget	taget	taget	taget	taget
NEUROD2		taget			taget			taget	taget
NGFR		taget			taget				
NMB		taget						taget	taget
NR4A1	taget				taget	taget		taget	taget
NRG2				taget	taget	taget		taget	taget
NRP2		taget		taget	taget			taget	taget
OAS1				taget		taget			taget
ODZ2		taget		taget					taget
PALLD	taget	taget							
PELI2								taget	
PERP				taget	taget			taget	
PGF	taget		taget	taget	taget	taget		taget	taget
PHLDA3	taget	taget		taget	taget	taget	taget	taget	taget
PIK3R1	taget	taget	taget	taget	taget		taget	taget	taget
PLA2G4A	taget	taget	taget	taget	taget	taget	taget	taget	taget
PLAU	taget	taget		taget		taget	taget	taget	taget
PLCD1	taget	taget		taget	taget	taget		taget	taget
PLEK2				taget	taget				
PTGS2	taget	taget	taget	taget	taget	taget	taget	taget	taget
PYGB									taget
RAB38								taget	taget
RAB9A					taget	taget		taget	taget
RAP1GDS1				taget	taget	taget		taget	taget
RASD1	taget								
RBPMS	taget	taget		taget	taget			taget	taget
RHOB	taget	taget	taget	taget	taget	taget		taget	taget
RPS27L				taget	taget			taget	
S100A2				taget	taget		taget	taget	taget
S100A6	taget	taget		taget			taget		
S100A9				taget	taget			taget	
SERPINB2	taget	taget	taget	taget		taget	taget	taget	taget
SERPINB5				taget	taget	taget	taget	taget	
SERPINE1	taget	taget	taget	taget	taget	taget	taget	taget	taget
SERPING1	taget	taget	taget	taget	taget	taget		taget	taget
SFN			taget	taget	taget		taget	taget	taget
SFRS11			taget			taget			taget
SLC30A6				taget	taget				
SLC31A2					taget				
SMURF2	taget				taget				
SNAI2						taget			taget
SNRPN								taget	
SPRR1A	taget	taget	taget	taget	taget		taget		
SPRR1B	taget	taget			taget	taget	taget	taget	
SPRR3	taget				taget				
SPTB	taget	taget			taget	taget		taget	taget
TAGLN3								taget	
TBC1D22A	taget								
TCF4				taget		taget		taget	taget
TFPI2	taget	taget	taget	taget	taget		taget		taget
THBS1				taget	taget		taget	taget	
TIMP1	taget	taget		taget	taget	taget	taget	taget	taget
TLE4	taget	taget			taget	taget		taget	taget
TNC	taget	taget	taget	taget	taget	taget	taget	taget	taget
TOB1	taget	taget	taget	taget	taget	taget	taget	taget	taget
TP53INP1				taget	taget				taget
TP63	taget	taget	taget	taget	taget	taget	taget	taget	taget

TRIM29	taget	taget							
UPK1A			taget	taget	taget			taget	taget
USP9X				taget	taget			taget	taget
VAMP3		taget		taget	taget			taget	taget
VASP				taget	taget	taget		taget	taget
VIM	taget	taget	taget	taget	taget		taget	taget	
VPS28	taget							taget	
XG	taget								
XPC				taget	taget			taget	

Differentailly over-expressed genes in mt p53 HNSCC cell lines

Gene symbol	RelA	cRel	NFκB1	p53	CEBPB	STAT3	AP1	EGR1	SP1
AARS			taget						
ABCC2	taget								
ABCC5	taget								
ABLIM1	taget								
AGPAT2							taget		
AGR2	taget								
AKR1B10	taget		taget						
AKR1C1	taget		taget		taget	taget		taget	
AKR1C2	taget		taget		taget	taget		taget	
AKR1C3	taget		taget		taget	taget		taget	
ALDH1A1	taget								
ALDH1A2		taget	taget		taget	taget		taget	
ALDH1A3	taget		taget				taget		
ALDH3A2							taget		
ANK1			taget				taget		
ANK3			taget						
AP3M2	taget	taget	taget	taget	taget	taget	taget	taget	taget
APOL1			taget						
ARID1A	taget	taget	taget	taget	taget	taget	taget	taget	taget
ARTS-1		taget	taget						
ATAD2		taget	taget		taget	taget	taget	taget	
ATF3	taget	taget	taget	taget	taget	taget			taget
ATP1B3			taget	taget			taget		taget
AXIN1			taget			taget			
BAG2			taget						
BATF			taget						
BCAN			taget					taget	
BMP7							taget		
BRF2							taget		
BRMS1			taget						
C15ORF48			taget						
C1S		taget	taget		taget	taget	taget	taget	
CA2	taget						taget		
CALML3			taget			taget			taget
CCND1			taget						
CDKN2C			taget		taget	taget		taget	taget
CEACAM5	taget		taget	taget			taget	taget	taget
CEBPA	taget								
CFB			taget						
CHEK2		taget	taget		taget	taget	taget	taget	
CHKA	taget		taget	taget		taget	taget	taget	taget
CIAO1		taget	taget		taget				
CIT			taget						
CLDN3	taget								
CLDN7			taget						
CLEC4E		taget				taget			
CNTNAP2							taget		

CP		taget	taget		taget	taget		taget	
CRISPLD1	taget								
CXCL14			taget	taget					taget
CYP26A1			taget			taget			
DCLK1			taget						
DGKG	taget				taget	taget		taget	
DLX5	taget		taget						
DNAJC4	taget		taget	taget		taget	taget	taget	taget
DYNC1I1	taget								
EIF4G1	taget	taget				taget		taget	
ELF3	taget	taget	taget	taget	taget	taget	taget	taget	taget
EPB41L4B			taget						
ERBB3	taget		taget	taget		taget	taget	taget	taget
ETV1		taget	taget		taget	taget	taget	taget	
FABP4		taget	taget		taget	taget		taget	
FABP5L7			taget				taget		taget
FADD							taget		taget
FBLN1						taget			
FBXL11	taget	taget	taget	taget	taget	taget	taget	taget	taget
FNTA	taget		taget	taget		taget	taget	taget	taget
FXYD3	taget						taget		
FZD1	taget		taget		taget	taget	taget	taget	taget
GDF5			taget			taget			
GLE1							taget		
GPNMB						taget		taget	
GPR1	taget								
GPR109B							taget		taget
GPR56			taget				taget		
GPX2	taget		taget		taget				
GSTA3			taget			taget			
GSTA4	taget		taget						
H1F0		taget							
HAP1	taget		taget	taget		taget	taget	taget	taget
HBE1			taget						
HCG		taget	taget	taget	taget				
HIST1H1C	taget	taget	taget	taget	taget	taget	taget	taget	taget
HLA-C						taget			
HLA-DMA			taget			taget			
HLA-DMB			taget			taget			
HLA-DPB1			taget			taget			
HLA-DRA			taget	taget	taget	taget	taget		taget
HLA-DRB1						taget			
HLA-E		taget				taget			
HLA-F			taget			taget			
HLA-G						taget			
HOXB7	taget		taget	taget		taget	taget	taget	taget
HOXD10			taget						
HRASLS3			taget						
ID3		taget	taget		taget	taget		taget	
IFITM1		taget			taget				
IFT172						taget			
IGF2	taget		taget	taget		taget	taget	taget	taget
IGFBP3	taget	taget	taget	taget	taget	taget	taget	taget	taget
IGFBP6			taget				taget		taget
IL22RA1		taget							
IL2RA	taget	taget	taget	taget	taget	taget	taget	taget	taget
IL6	taget	taget	taget	taget	taget	taget	taget	taget	taget
IMPA2			taget						
INA	taget			taget			taget	taget	taget

ISG15		taget		taget	taget	taget	taget	taget	
KCNC1			taget	taget		taget		taget	taget
KCND2	taget		taget	taget	taget	taget	taget	taget	taget
KLK10								taget	taget
KRT1	taget								
KRT4	taget					taget	taget		
LAMP3			taget			taget			
LCN2		taget			taget	taget	taget		
LDB3							taget		
LHX2			taget						
LIMCH1	taget								
JMJD7			taget						
LY6D	taget	taget	taget			taget	taget	taget	taget
MAGEF1	taget								
MAP2K5			taget						
MATN2			taget	taget			taget		taget
MCCC1			taget						
MEIS2	taget	taget	taget	taget	taget	taget	taget	taget	taget
MMP1	taget		taget	taget	taget		taget		taget
MMP13			taget				taget		
MRPL21			taget					taget	
MSLN	taget								
MUC1	taget		taget	taget		taget	taget	taget	taget
MYO5B		taget				taget			
MYST3							taget		
NFE2L2	taget	taget	taget	taget	taget	taget		taget	taget
NFIL3		taget	taget		taget	taget	taget	taget	
NOV						taget			
NQO1	taget		taget			taget	taget	taget	taget
NTRK2	taget		taget			taget	taget	taget	taget
OAS2		taget		taget	taget		taget	taget	
P2RY5			taget						
PALMD		taget							
PDZK1IP1		taget				taget			
PLCG2							taget		taget
PLEKHF2		taget							
PMS2	taget	taget	taget	taget	taget	taget	taget	taget	taget
POPDC3	taget								
PPAP2C						taget			
PPFIA1			taget						
PRG3	taget								
PRODH	taget	taget	taget	taget	taget	taget	taget	taget	taget
PROSC							taget		
PSMB7							taget		
PSORS1C2			taget			taget		taget	
PTGES	taget	taget		taget	taget	taget	taget	taget	taget
PTGS1							taget		
PTK2	taget	taget	taget	taget	taget	taget	taget	taget	taget
PTPN7	taget	taget	taget	taget	taget	taget	taget	taget	taget
PTPRN2			taget						
PVRL1	taget		taget		taget	taget		taget	
RAB22A			taget						
RAB3B	taget								
RABGAP1			taget						
RALGDS							taget		
RARRES3		taget					taget		
RASL10A							taget		
RBM13			taget				taget		
REL	taget		taget	taget	taget	taget		taget	taget

RGS2	taget		taget						
RPS6KA5			taget						
RREB1	taget								
RYR1			taget						
SAA1	taget	taget	taget	taget	taget	taget	taget	taget	taget
SCGB1A1			taget			taget			
SCHIP1	taget		taget				taget		
SCNN1A	taget		taget		taget			taget	
SERPINF1	taget	taget	taget	taget	taget		taget	taget	taget
SERPINI1	taget		taget			taget			
SEZ6L2	taget		taget	taget	taget	taget	taget	taget	taget
SIRT7			taget						
SNCG	taget								
SPP1	taget	taget	taget	taget	taget	taget		taget	taget
SPRR2A			taget			taget		taget	
TAF15	taget		taget		taget			taget	
TAPBP		taget				taget			
TDRD7			taget						
TGM1			taget						
TNFAIP2						taget			
TNFSF10	taget		taget			taget			
TOP2A			taget						
TSPAN13						taget			
UBE2C			taget						
UGT1A6	taget						taget	taget	
ULBP2			taget				taget		
VIPR1			taget						
VSNL1	taget	taget				taget		taget	
XAF1		taget				taget			
XCL1			taget						
ZNF14			taget						
ZNF266							taget		

Differentailly under-expressed genes in mt p53 HNSCC cell lines

Gene symbol	RelA	cRel	NFκB1	p53	CEBPB	STAT3	AP1	EGR1	SP1
ACLY	taget	taget	taget	taget	taget	taget	taget	taget	taget
ACSL3	taget								
ACSS2							taget		
ACTB	taget								
ACTN3		taget							
ACTR1A		taget							
ADAM8						taget		taget	taget
ADAMTS1	taget								
AKT1	taget	taget				taget	taget	taget	taget
ANGPT1	taget	taget	taget	taget	taget		taget	taget	taget
ANXA3	taget						taget		
AOX1	taget								
AREG	taget						taget		
ARHGEF10	taget								
ARL7	taget								
ARMET	taget								
ATP10D		taget							
ATP2A2	taget	taget			taget			taget	taget
B4GALT1	taget				taget		taget	taget	taget
BCAP29	taget								
BCAR3	taget	taget							
BCL11B	taget	taget	taget	taget	taget	taget		taget	taget
BMI1	taget	taget			taget	taget	taget	taget	taget
BNC1	taget	taget			taget	taget	taget	taget	taget

C20ORF10	taget								
C21ORF56	taget								
C9ORF46	taget								
CA12	taget	taget							
CAB39	taget								
CALD1	taget								
CALU	taget	taget				taget	taget	taget	taget
CAP1	taget								
CAPN2						taget			
CD59	taget								
CD96	taget								
CDA	taget								
CDC42EP2	taget								
CDH13		taget							
CDK5R1	taget	taget	taget	taget	taget	taget		taget	taget
CDKN1A	taget	taget	taget	taget	taget	taget	taget	taget	taget
CENTA1	taget						taget		
CKAP4	taget				taget		taget	taget	taget
CLCA2		taget							
COL12A1	taget	taget			taget			taget	taget
COL8A1	taget								
CRIM1	taget	taget			taget		taget		taget
CSF2	taget	taget	taget	taget	taget	taget	taget	taget	taget
CTRB2	taget								
CTSB	taget								
CTSC	taget								
CYP3A4	taget	taget			taget				
CYR61	taget								
DAPP1	taget								
DGKA	taget	taget	taget		taget	taget	taget	taget	taget
DISC1	taget								
DKK3	taget						taget		
DNAJB5	taget	taget	taget		taget		taget	taget	taget
DNAJC15						taget			
DNTTIP2	taget								
DUSP4	taget								
DUSP5		taget	taget	taget	taget	taget	taget		taget
DUSP6	taget								
EDN1		taget							
EFNB1	taget	taget							
EGLN1	taget	taget							
ELL2	taget								
EMB		taget							
EMP1		taget							
ENC1	taget	taget	taget	taget	taget	taget	taget	taget	taget
ERCC1	taget						taget		
EREG	taget								
ESDN	taget								
ETHE1							taget		
ETS1	taget								
ETV4	taget	taget	taget		taget	taget	taget	taget	taget
EXTL3	taget								
F2RL1	taget								
FADS2	taget								
FASTK	taget								
FBN2	taget					taget	taget	taget	taget
FDFT1	taget								
FDPS	taget								
FEZ1	taget					taget			

FGFBP1	taget	taget	taget		taget	taget			taget
FHL1	taget						taget		
FILIP1L	taget								
FJX1	taget								
FKBP5	taget								
FLRT2		taget							
FMO3	taget								
FN1	taget								
FOLR3	taget								
FOSL1		taget							
FOXD1	taget								
FST	taget	taget			taget		taget	taget	taget
FSTL3	taget								
G0S2	taget	taget		taget	taget		taget	taget	taget
GATA3		taget					taget		
GCG	taget					taget	taget	taget	taget
GFRA3		taget							
GHRH						taget			
GJB3		taget				taget			
GJB5	taget	taget			taget	taget		taget	taget
GNA15		taget							
GNB2	taget	taget	taget	taget	taget	taget	taget	taget	taget
GNB4		taget							
GNG12	taget								
GPX1	taget		taget	taget	taget	taget	taget	taget	taget
GTF2H4	taget								
GZF1	taget	taget							
HERC3	taget								
HMGCR	taget								
HR	taget	taget	taget	taget	taget	taget	taget	taget	taget
ID1	taget								
IL1A	taget	taget	taget	taget		taget		taget	taget
IL1B	taget	taget	taget	taget	taget	taget	taget	taget	taget
IL1R2	taget	taget	taget				taget		taget
IL4R	taget								
INHBA		taget						taget	
IRF5	taget			taget	taget		taget	taget	taget
ITGA2						taget	taget	taget	
ITGA3	taget	taget	taget	taget	taget	taget	taget	taget	taget
ITGA5	taget	taget	taget	taget	taget	taget	taget	taget	taget
ITGB4	taget	taget			taget	taget		taget	taget
ITGB6	taget	taget		taget		taget			
KLF6	taget								
KLF7	taget	taget	taget	taget	taget	taget		taget	taget
KRT31	taget								
KRT6B		taget							
LAMA3	taget	taget	taget	taget		taget	taget		taget
LAMB3	taget	taget	taget	taget		taget	taget	taget	taget
LAMC2	taget	taget	taget			taget			taget
LIMA1	taget						taget	taget	
LIPG	taget						taget		
LOC100128809	taget	taget							
LOC100132916	taget								
LOC729948	taget								
LPIN1	taget								
LTBP2			taget						
MAFK	taget								
MALT1	taget								
MAP4K4	taget	taget		taget	taget		taget	taget	taget

MAPKAPK3	taget								
MBD1	taget								
MCAM	taget								
MFAP2	taget								
MFAP5	taget		taget			taget	taget	taget	
MGLL	taget	taget	taget		taget		taget	taget	taget
MLL	taget		taget	taget	taget	taget	taget	taget	taget
MPZL2		taget							
MRPL12	taget	taget							
MST1R	taget					taget	taget	taget	
MT1E		taget					taget		
MT1F	taget								
MT1G	taget								
MT1L	taget						taget		
MT1X		taget							
MT2A	taget								
MYC	taget	taget	taget	taget	taget	taget	taget	taget	taget
MYH3	taget	taget			taget		taget	taget	taget
MYH9		taget				taget			
MYO1E	taget								
NCOA5	taget					taget	taget	taget	taget
NDRG1	taget	taget	taget	taget	taget	taget	taget	taget	taget
NR4A1	taget	taget		taget		taget	taget	taget	taget
NRP2	taget	taget		taget	taget		taget	taget	taget
NT5DC3	taget								
NT5E	taget								
OAT		taget							
OSBPL10	taget								
OXSR1	taget								
PALLD	taget								
PANX1	taget								
PAPSS1	taget								
PBX3	taget								
PCMTD2		taget							
PELI2		taget				taget			
PFKP	taget								
PGF	taget	taget			taget	taget	taget	taget	taget
PHLDA1	taget								
PITRM1	taget								
PLA2G7	taget								
PLAU	taget	taget	taget	taget		taget	taget	taget	taget
PLCD1	taget	taget			taget	taget	taget	taget	taget
PLEK2	taget					taget	taget	taget	
PLK3	taget	taget	taget	taget	taget	taget		taget	taget
PMEPA1	taget								
POU3F4		taget							
PPP1R3D	taget	taget	taget	taget	taget	taget	taget	taget	taget
PRO1933	taget								
PSG1	taget								
PTGS2	taget	taget	taget	taget	taget	taget	taget	taget	taget
PTH2R		taget							
PTHLH	taget	taget			taget	taget		taget	taget
PVALB		taget					taget		
RAB38	taget				taget		taget	taget	taget
RAC2	taget								
RAP1GDS1	taget	taget			taget			taget	taget
RASD1	taget								
RBPMS	taget	taget			taget		taget	taget	taget
RGS20	taget					taget			

RHOA	taget	taget	taget				taget	taget	taget
RHOC	taget								
RNH1	taget								
RPL22L1	taget								
RPS27L	taget	taget	taget	taget	taget	taget	taget	taget	taget
S100A2	taget	taget	taget	taget	taget	taget	taget	taget	taget
S100A6	taget	taget	taget		taget	taget	taget		taget
S1PR2	taget								
SAT1	taget								
SC4MOL							taget		
SEC22C	taget								
SEPP1		taget					taget		
SERPINB2		taget	taget		taget	taget	taget	taget	taget
SERPINB5	taget		taget	taget	taget	taget		taget	taget
SERPINB7	taget								
SERPINE1	taget	taget	taget	taget	taget	taget	taget	taget	taget
SERPING1	taget					taget	taget	taget	taget
SFN	taget	taget	taget	taget	taget	taget	taget	taget	taget
SFRP1	taget								
SH3KBP1	taget	taget	taget		taget	taget	taget	taget	taget
SHC1	taget								
SLC16A2	taget								
SLC20A1	taget								
SLC2A3							taget		
SLC30A6	taget					taget			
SLMAP	taget								
SMURF2	taget								
SNAI2		taget					taget	taget	
SORBS3	taget								
SOX9	taget								
SPARC		taget							
SPTB	taget	taget	taget				taget	taget	taget
SQLE	taget								
SRPX	taget								
TAGLN3	taget								
TBX3	taget	taget	taget	taget	taget	taget	taget	taget	taget
TCF4	taget	taget	taget	taget	taget	taget		taget	taget
TFPI2	taget	taget	taget		taget	taget	taget	taget	
TGFBR2	taget								
THBS1	taget	taget	taget	taget	taget	taget	taget	taget	taget
TIMP1	taget		taget		taget	taget	taget	taget	taget
TM7SF3	taget								
TMOD3	taget								
TMSB10		taget							
TNFRSF6B	taget		taget				taget	taget	
TOB1	taget	taget	taget	taget	taget	taget	taget	taget	taget
TSPAN1	taget								
TSPAN5	taget								
UAP1	taget								
UPP1		taget						taget	
VASP	taget	taget			taget	taget	taget	taget	taget
VDR		taget							
VEGFC	taget								
VIM	taget	taget	taget		taget	taget	taget	taget	
VPS28	taget	taget							
WDR1	taget								
XG	taget					taget			
