# Supplementary material for: Unraveling Regulatory Programs for NF-kappaB, p53 and MicroRNAs in Head and Neck Squamous Cell Carcinoma
Source: PLoS One. 2013 Sep 19;8(9):e73656. doi: 10.1371/journal.pone.0073656 (PMC3777940; doi:10.1371/journal.pone.0073656)
Supplement: Table S2 — Overlapping of target genes between NF-κB or p53 and other TFs in HNSCC cell lines. (PDF) [file pone.0073656.s003.pdf]

	NF-κB	RelA	NFκB1	cRel	p53	CEBPB	STAT3	AP1	EGR1	SP1
wt-over total [a]	149	71	79	99	73	75	84	94	64	69
NF-κB [b]		71	79	99	42	**66**	**65**	50	**47**	39
p53 [c]	42	26	27	**37**		**33**	**34**	30	**38**	**37**
wt-under total [a]	99	75	47	71	96	99	72	51	99	106
NF-κB [b]		75	47	71	**60**	**69**	**49**	**48**	**61**	**69**
p53 [c]	**60**	**46**	**39**	**53**		**77**	**50**	**41**	**64**	**70**
mt-over total [a]	169	78	126	49	41	49	86	75	64	50
NF-κB [b]		78	126	49	**41**	**49**	**76**	**58**	**62**	**46**
p53 [c]	**41**	**33**	**37**	**22**		**27**	**32**	**34**	**34**	**38**
mt-under total [a]	249	213	52	109	40	64	74	86	81	77
NF-κB [b]		213	52	109	**40**	**64**	**69**	**81**	**79**	**76**
p53 [c]	**40**	**39**	**34**	**36**		**34**	**35**	**32**	**37**	**39**

[a] total target gene number of the nine TFs (RelA, NFκB1, cRel, p53, AP1, CEBPB, EGR1, SP1 and STAT3) in the over- or under-expressed subset of wt p53-deficient or mt p53 tumor cell lines. NF-κB target genes include those of at least one of its three subunits RelA, NFκB1 and cRel. [b] target gene numbers overlapped with at least one of three NF-κB subunits. [c] target gene numbers overlapped with p53. Number in bold refers to significant overlap between two gene groups (P<0.001). The statistical significance of the overlaps between different gene sets was calculated based on hypergeometric distribution.
