# Supplementary material for: Unraveling Regulatory Programs for NF-kappaB, p53 and MicroRNAs in Head and Neck Squamous Cell Carcinoma
Source: PLoS One. 2013 Sep 19;8(9):e73656. doi: 10.1371/journal.pone.0073656 (PMC3777940; doi:10.1371/journal.pone.0073656)
Supplement: Table S3 — Significant enrichment of biological functions among common target genes between NF-κB and p53 in HNSCC cell lines. Joint target genes of NF-κB and p53 were annotated to, A. GO biological processes by using DAVID tool with P<0.001, FDR <2%. B, Canonical signaling pathways by using Ingenuity Pathway Analysis tools (P<0.05). Genes in red and green indicate the over- and under-expressed genes in HNSCC cell lines above at least 2 fold changes, respectively. (PDF) [file pone.0073656.s004.pdf]

A. GO biological processes by using DAVID tool

Biological process	Gene no.	FDR	P value
wt p53-deficient HNSCC cell lines			
regulation of cell proliferation	26	3.54E-08	2.16E-11
regulation of apoptosis	20	0.00146598	8.97E-07
epithelium development	11	0.003791	2.32E-06
epidermis development	9	0.04539463	2.78E-05
angiogenesis	8	0.08662668	5.30E-05
immune system development	10	0.13415124	8.21E-05
inflammatory response	10	0.45775321	2.81E-04
apoptosis	13	0.90795101	5.58E-04
regulation of cell migration	7	1.394923	8.59E-04
hemopoiesis	10	0.03970911	2.43E-05
hemopoietic or lymphoid organ development	10	0.0846974	5.18E-05
blood vessel morphogenesis	9	0.11987483	7.34E-05
positive regulation of cell proliferation	12	0.13477731	8.25E-05
vasculature development	9	0.39638367	2.43E-04
positive regulation of transport	8	1.07338585	6.60E-04
response to hormone stimulus	10	1.10271133	6.78E-04
mt p53 HNSCC cell lines			
regulation of cell proliferation	23	2.25E-07	1.37E-10
regulation of apoptosis	21	1.41E-05	8.56E-09
cell motion	14	0.00298275	1.81E-06
cell migration	11	0.00449995	2.74E-06
regulation of inflammatory response	7	0.0062673	3.81E-06
regulation of cell cycle	11	0.02233142	1.36E-05
positive regulation of transcription, DNA-dependent	12	0.09775177	5.95E-05
inflammatory response	10	0.1222542	7.44E-05
positive regulation of gene expression	13	0.12680424	7.71E-05
angiogenesis	7	0.27536743	1.68E-04
positive regulation of transcription	12	0.42723324	2.60E-04
blood vessel development	8	0.66964595	4.08E-04
positive regulation of cell communication	9	0.75356379	4.60E-04
regulation of cytokine production	7	0.81106051	4.95E-04
regulation of cell adhesion	6	1.58085307	9.68E-04
positive regulation of RNA metabolic process	12	0.10537934	6.41E-05
response to hormone stimulus	10	0.30675118	1.87E-04
positive regulation of protein kinase cascade	7	0.52877374	3.22E-04
vasculature development	8	0.77381339	4.72E-04
response to steroid hormone stimulus	7	1.10530962	6.75E-04
regulation of cell development	7	1.55202419	9.50E-04
anti-apoptosis	7	1.59134711	9.75E-04

B. Signaling pathways by using Ingenuity Pathway Analysis (IPA) tool

Ingenuity Canonical Pathways	cell line	Genes in the pathway
CDK5	mt	CDK5R1, ITGA3, PPP1R3D
HER-2 in Breast Cancer		**CDKN1A** , ERBB3 , ITGB6
Protein Kinase A		DUSP5, GNB2, HIST1H1C , PPP1R3D, **PTGS2,** **PTK2** , PTPN7 , SFN, TCF
Small Cell Lung Cancer		MYC, **PTGS2,** **PTK2**
14-3-3-mediated	wt	PIK3R1, PLCD1, SFN, VIM
HIF1α		MMP1 , PGF, PIK3R1
Leukocyte Extravasation		CLDN4 , ICAM1 , MMP1 , PIK3R1, TIMP1
mTOR		EIF4G1 , PGF, PIK3R1, RHOB
NF-κB		BMP4, IL1A, IL1R2, IL1RN, PIK3R1
TGFβ		BMP4, INHBA, **SERPINE1**
VEGF		PGF, PIK3R1, SFN
Xenobiotic Metabolism		ALDH1A3 , IL1A, **IL6** , NQO1 , PIK3R1
Ephrin Receptor	mt	ANGPT1, GNB2, ITGA3, ITGA5, MAP4K4, **PTK2**
	wt	ANGPT1, ITGA3, ITGA5, PGF
ERK/MAPK	mt	ELF3 , ITGA3, ITGA5, MYC, PPP1R3D, **PTK2**
	wt	ELF3 , ITGA3, ITGA5, PIK3R1, PLA2G4A
HGF	mt	**CDKN1A** , ELF3 , **IL6** , **PTGS2,** **PTK2**
	wt	**CDKN1A** , ELF3 , **IL6** , PIK3R1, **PTGS2**
IGF-1	mt	IGFBP3 , **PTK2** , SFN
	wt	IGFBP3 , PIK3R1, SFN
IL-6	mt	IL1A, IL1B, **IL6** , MAP4K4
	wt	IL1A, IL1R2, IL1RN, **IL6** , IL8 , PIK3R1
IL-8	mt	ANGPT1, GNB2, MAP4K4, **PTGS2,** **PTK2**
	wt	ANGPT1, ICAM1 , IL8 , PGF, PIK3R1, **PTGS2,** RHOB
Integrin Linked Kinase	mt	ITGB6, MUC1 , MYC, **PTGS2,** **PTK2**
	wt	KRT18 , PGF, PIK3R1, **PTGS2,** RHOB, VIM
Integrin	mt	ITGA3, ITGA5, ITGB6, **PTK2**
	wt	ITGA3, ITGA5, PIK3R1, RHOB
Neuregulin	mt	CDK5R1, ERBB3 , ITGA3, ITGA5, MYC
	wt	ITGA3, ITGA5, PIK3R1
p38 MAPK	mt	IL1A, IL1B, MYC
	wt	IL1A, IL1R2, IL1RN, PLA2G4A
p53	mt	**CDKN1A** , SERPINB5, SFN, THBS1
	wt	**CDKN1A** , PIK3R1, SFN, **TP63**
PI3K/AKT	mt	**CDKN1A** , ITGA3, ITGA5, **PTGS2,** SFN
	wt	**CDKN1A** , ITGA3, ITGA5, PIK3R1, **PTGS2,** SFN
PPAR	mt	IL1A, IL1B, MAP4K4, **PTGS2**
	wt	IL1A, IL1R2, IL1RN, **PTGS2**
PTEN	mt	**CDKN1A** , ITGA3, ITGA5, **PTK2**
	wt	**CDKN1A** , ITGA3, ITGA5, PIK3R1
TREM1	mt	CSF2, IL1B, **IL6** , ITGA5
	wt	CASP1 , CSF2, ICAM1 , **IL6** , IL8 , ITGA5
