# Supplementary material for: Unraveling Regulatory Programs for NF-kappaB, p53 and MicroRNAs in Head and Neck Squamous Cell Carcinoma
Source: PLoS One. 2013 Sep 19;8(9):e73656. doi: 10.1371/journal.pone.0073656 (PMC3777940; doi:10.1371/journal.pone.0073656)
Supplement: Table S4 — Target genes of mir21 and mir34ac and their overlapping with NF-κB and p53 targets in HNSCC cell lines. Based on miRNA databases TarBase and mir2disease, “target validated” refers to target genes of mir21 or mir34ac tested by reporter assay, and “target likely” refers to genes of mir21 or mir34ac tested by other methods (such as microarray, qRT-PCR, Western blot, and proteomics). The number in the parenthesis refers to number of computational methods (≥3) for the miRNA target gene prediction based on miRecords. The genes in red and green represent differentially over- and under-expressed at least fold change 2.0 in the wt and mt p53 cells. (PDF) [file pone.0073656.s005.pdf]

wt p53-deficient HNSCC cell lines

Gene symbol	RelA	NFκB1	cRel	p53	mir21	mir34ac
AARS						target
ACSL1		target		target	target	target validated(6)
ACSL4				target	target	target validated(6)
AGER	target	target	target	target	target	target
ALDH1A3	target				target	
ANXA3					target	
ANXA8L1					target	target
ASS1	target	target	target	target	target	target
BIRC2		target	target		target	
BTG2	target	target	target	target	target validated(5)	target(3)
C20ORF24	target	target	target	target		target
CASP1	target			target	target	target
CASP4						target
CDKN1A	target	target	target	target	target likely	target
CLDN3			target		target	
COL12A1				target	target(3)	target(6)
CSF1	target	target	target	target		target
CSF2	target	target	target	target	target	target
DDEF1					target	target
DHX35						target(3)
DIO2	target	target	target			target
DNAH11						target
DSG3					target(3)	target
DUSP14	target	target	target	target	target	target
EHD1				target	target	target likely
ELF3	target	target	target	target		target
EPPB9						target
ETV1	target				target(3)	
FAM115A					target	
FUT8	target	target	target	target		target(6)
GJB2					target	
GPR109B			target	target	target	
GPX2				target	target	
GULP1						target
HLA-A	target	target	target	target	target	target
HTATIP2						target
ICAM1	target	target	target	target	target	target(3)
IFI27				target		target
IGFBP3	target		target	target		target(4)
IGFBP6						target
IKBKE	target	target	target			target(4)
IL1A	target	target	target	target	target	target
IL1R2	target	target	target	target		target
IL1RN	target	target	target	target	target	target(3)
IL6	target	target	target	target	target	target
IL8	target	target	target	target	target	target(3)
IRF4	target	target	target	target	target	target(3)
ITGA3				target		target(3)
ITGA5	target	target	target	target	target	target
ITGA6				target		target likely(3)
JAG1		target	target	target	target validated(6)	target validated(5)
KRT14		target				target
KRT19			target		target	
LAMA3		target			target	

LAMP3					target	target
LCN2		target				target
LIMA1						target(3)
MFAP5					target(3)	
MGST1			target		target	
MMP1	target	target	target	target	target	target
MUC4			target		target	
NDRG1		target		target	target	target likely(4)
NDUFB9					target	
NMB					target	target
NQO1	target	target	target	target		target(4)
ODZ2					target	target
PALM						target
PARP3	target		target		target	
PCBP2				target	target(3)	target
PERP		target		target	target	target
PHLDA3	target	target	target	target	target	target
PIK3R1						target
PLA2G4A	target	target	target			target
PLAU	target	target	target	target	target	target(3)
PLEK2						target
PPAP2C						target(3)
PTGES	target		target	target	target	target
PTGS2	target	target	target	target	target	target(3)
PTPRM				target	target	target(4)
RHOB					target likely(5)	target
RPN2	target				target	
RPS27L	target	target	target	target	target	target
S100A2		target	target	target	target	target
S100A6	target	target	target	target	target	target
SCNN1A	target	target	target	target		target(3)
SERPINB5		target		target	target validated	target
SERPINE1	target	target	target	target	target	target(5)
SFN				target	target	target likely
SFRS11					target likely	
SLC2A4RG	target		target			target(6)
SNAI2						target
SPRR1B					target	
STC1	target	target	target	target	target(3)	target(5)
SYT17						target
TGFBI	target	target	target	target	target likely(6)	target likely(3)
TGFBR2				target	target validated(4)	target validated(3)
THBS1				target	target likely(3)	target likely
TMEM123	target				target	
TNC	target	target	target	target	target	target
TOB1				target		target
TP53INP1	target	target	target	target	target(3)	target(3)
TP63	target	target		target		target(3)
TRIM29						target
UPK1A				target	target	target
VGLL1	target		target		target(3)	
WFDC2						target
XPC	target	target	target	target	target	target(3)
YAP1	target		target	target	target(6)	

mt p53 HNSCC cell lines

Gene symbol	RelA	NFκB1	cRel	p53	mir21	mir34ac

ACSL4	target	target	target	target	target	target validated(6)
ACSS2						target
ACTR1A	target	target	target		target	target(4)
ADAM8						target
ALDH1A3					target	
ANK1		target			target	
ARID1A	target		target	target	target	target(4)
ATF3	target	target	target	target	target	target
ATP10D						target
ATP1B3				target		target(3)
BMP7					target	
C20ORF24	target		target	target	target	target
CA2					target	
CALML3	target					target(3)
CDKN1A	target	target	target	target	target likely	target
CNTNAP2		target	target	target	target	target(5)
COL12A1	target	target	target	target	target(3)	target(6)
CRIM1	target	target		target	target(5)	target(3)
CSF2	target	target	target	target	target	target
CXCL14		target		target		target(3)
DUSP5		target	target	target	target(3)	target
EHD1	target	target		target	target(4)	target likely
ELF3	target	target	target	target	target	target
ENC1	target	target	target	target	target(3)	target
FGFBP1	target		target		target	
FNTA					target	
FXYD3						target
GCG						target
GJB5					target	
GPR109B		target			target	target
GPX1	target	target	target	target	target	target
GPX2	target		target		target	
GZF1			target			target
HLA-DRA		target		target	target	target
IGFBP3	target	target	target	target	target(3)	target(4)
IGFBP6					target	target
IL1A	target	target	target	target	target	target
IL1B	target	target	target	target	target(3)	target
IL1R2	target	target	target			target
IL2RA	target	target	target	target	target	target
IL6	target	target	target	target	target	target
ITGA3		target	target	target	target	target(3)
ITGA5	target	target	target	target	target	target
ITGB4					target	
ITGB6					target	
KCNC1	target	target				target
KCNH2		target	target	target	target	target(3)
KLK10				target		target(3)
KRT4	target				target	
LAMA3		target		target	target	target
LAMB3	target	target	target	target	target	target
LAMC2	target	target			target	target(3)
LCN2			target			target
LY6D	target	target	target		target	target
MATN2			target	target	target(6)	target
MFAP5		target	target		target(3)	target
MMP1	target	target	target		target	target

MMP13		target			target	target
MYC	target	target	target	target	target likely	target validated(3)
NDRG1	target	target	target	target	target	target likely(4)
PEA15	target	target	target	target	target	target likely(6)
PELI2					target(4)	
PGM1	target	target	target	target	target(3)	target(6)
PLAU	target	target	target	target	target	target(3)
PLCG2					target	
PLK3	target	target	target	target	target	target
PMS2	target	target	target	target	target	target
PPFIA1			target	target	target	target(4)
PRODH			target	target	target	target
PSORS1C2		target			target	
PTGES		target	target	target	target	target
PTGS1					target	
PTGS2	target	target	target	target		target(3)
PTK2	target	target	target	target	target likely	
PTPRM	target	target	target		target	target(4)
RAB38						target
RALGDS			target	target	target	target(4)
RBM13					target	
RPS27L	target	target	target	target	target	target
S100A2	target	target		target	target	target likely
S100A6	target	target	target	target		target
SAA1	target	target	target	target	target	target
SERPINB5	target	target	target	target	target validated	target
SERPINE1	target	target	target	target	target	target(5)
SERPINF1	target	target	target	target	target	target
SFN	target	target	target	target		target likely
SPP1	target	target	target	target		target(3)
TGFBR2	target	target		target	target validated(4)	target(3)
THBS1	target	target	target	target	target likely(3)	target
TOB1	target		target	target	target	target
UGT1A6	target	target				target
ULBP2					target	target(3)
VAT1	target	target	target		target	target(5)
ZCCHC3	target			target	target(5)	target(3)
ZNF266					target	
