# Supplementary material for: Unraveling Regulatory Programs for NF-kappaB, p53 and MicroRNAs in Head and Neck Squamous Cell Carcinoma
Source: PLoS One. 2013 Sep 19;8(9):e73656. doi: 10.1371/journal.pone.0073656 (PMC3777940; doi:10.1371/journal.pone.0073656)
Supplement: Table S5 — Overlapped target genes of NF-κB, p53, mir21 and mir34ac in HNSCC tissues. (Legends see Table S4). (PDF) [file pone.0073656.s006.pdf]

Oral cancer tissues from O'Donnell et al., GSE2280 (NCBI GEO accession number)

Gene symbol	RelA	NFκB1	cRel	p53	mir21	mir34ac
ALDOA	target	target		target	target	target(6)
ALOX12B	target	target	target			target
ARHGAP1	target			target	target	target likely(5)
ASS1	target	target	target	target	target	target
BCL2	target	target	target	target	target validated(4)	target validated(7)
BHMT	target	target	target	target	target	target
BMP4	target	target		target	target	target
CCL20	target	target			target(4)	
CCL5		target	target		target	target
CEP57			target	target	target	target
CLIP3	target				target	
COL1A2	target	target		target	target	target
CPEB3	target	target	target	target	target likely(5)	target(5)
CR2	target	target	target	target	target(3)	target(4)
CSF1	target	target		target	target	target
CXCR5	target	target	target	target	target(3)	target
DIO2	target	target			target(3)	target
DNAJC16	target	target	target	target	target(4)	target(4)
DSC3				target	target	
EHD1	target				target(4)	
ELF3	target	target	target		target	target
FAS	target	target	target	target	target likely	target(3)
FOS	target	target		target	target	
GNLY	target	target	target	target	target	target
GPR64	target	target	target	target	target likely(5)	target(4)
GPX1				target	target	
GUCY1A3	target				target	
GZMB	target	target	target	target	target	target
HLA-DPA1		target				target
HNRNPK	target			target	target likely(5)	target(3)
IER3	target	target	target	target	target	target
IFNB1	target	target	target	target	target	target likely
IL1B	target	target	target	target	target(3)	target
IL1R2	target	target			target	target
IL1RN	target	target	target		target	target(3)
IL2RA	target	target	target			target
IL6	target	target	target	target		target
IL8	target	target	target	target	target	target(3)
JMJD4			target		target	
KDELR3	target				target	
LRRC32	target				target	
MIIP	target				target	
MMP1	target	target		target	target	target
MMP9	target	target	target	target	target likely	target
NES	target				target(3)	
NFKB2	target	target	target	target	target	target
NOD2	target	target	target	target	target	target
NQO1	target	target			target	target(4)
PCBP1				target	target likely(5)	

PCNA				target	target	
PERP		target	target	target	target	target
PLA2G4A	target	target	target	target	target(3)	target
PLAU	target	target	target	target		target(3)
PSMA2	target	target		target	target	target(3)
PSMB9		target	target		target	target
PSME2		target				target
PTX3		target	target	target	target	target
RPS27L				target	target	
SCNN1A	target	target			target	target(3)
SELE	target	target	target	target	target	target
SEMA4C	target			target	target	target(5)
SERPINB5			target	target	target validated	
SERPINE1	target	target	target	target	target	target(5)
SERPINF1	target	target		target	target	target
SLC19A1	target				target	
SOD1		target				target
SOX2		target			target likely(5)	
STAT4	target	target	target	target	target	target
TAP1	target	target	target		target	target
TGFBI	target		target	target	target likely(6)	target(3)
TIMP3	target				target validated(6)	target
TNC	target	target	target	target	target	target
TP63	target	target	target	target	target validated(3)	target(3)
TPM1	target		target	target	target validated	target likely(3)
XRCC5				target	target	
ZNF747	target				target	

Hypopharyngeal cancer tissues from Cromer et al., GSE2379 (NCBI GEO accession number)

Gene symbol	RelA	NFκB1	cRel	p53	mir21	mir34ac
ACAA2			target			target
ADNP	target					target likely(3)
AKAP9	target				target likely	
ALDH4A1			target		target	
ALDH9A1	target					target likely
ALDOA	target	target	target	target	target	target(6)
ALG8	target					target
ALOX12B	target	target	target	target	target	target
AMOT				target		target(3)
ANP32A				target	target	
ASS1	target	target	target	target		target
ATP11B		target			target likely(3)	
ATP5G3			target		target	
BNIP3L			target		target(3)	
BTN3A2	target			target		target(3)
BUB1B	target				target	
C10ORF10		target			target	
CBX1	target				target	
CBX6		target	target		target	
CCDC69			target			target
CCL4	target		target	target	target	target

CD200				target	target	
CD247				target		target(3)
CD27			target			target
CD48	target	target		target	target(3)	target
CD70		target	target		target	
CDKN1B			target			target(3)
CENPF			target		target	
CFI				target	target	
CITED2	target					target
CLASP2	target					target(3)
CNPY3				target		target
CREM	target					target
CTGF	target				target	
CXCL1		target			target	
CYBB	target					target(4)
CYP1B1		target				target
DDIT4		target		target	target	
DEK		target	target		target	
DNAJA1	target					target
DOCK2			target			target
DTX4		target	target		target	
DUSP10	target				target	
DUXAP10			target		target	
DYRK1A	target					target(3)
ECM2	target					target
EFS				target	target	
EVI2B			target			target
FABP7				target	target	
FADS3			target		target	
FAM171A1		target				target
FARP1	target					target(3)
FBLN1	target				target	target
FCHSD2	target		target			target
FERMT2	target					target(3)
GPC4				target	target(4)	
GSTP1	target	target	target	target		target
GTPBP6		target	target		target	
GTSE1			target		target	
GZMK			target			target
HERPUD1	target					target
HES1				target	target	
HMGB2		target			target	
HMGN3			target		target	
HUWE1			target		target	
ICAM2			target			target(3)
IGFBP3		target	target	target	target(3)	target(4)
IL1B	target	target	target	target	target(3)	target
IL6	target	target	target	target	target	target
IL8	target	target	target	target	target	target(3)
IRF4	target	target	target	target	target	target(3)
JARID2		target	target		target	

KCNN4				target	target	
KDM5D				target		target
KIAA0907	target					target
LAMB1	target				target	
LAMB3	target	target	target	target	target	target
LBR	target				target	
LDHA	target	target	target	target	target	target validated(5)
LDOC1	target					target(3)
LEPROTL1	target					target(3)
LMO4	target					target
LOC729678			target			target
LTB4R			target		target	
LUC7L3	target					target
LYPD3			target		target	
MAGED1		target			target	
MAN2A2	target					target(3)
MCM3AP		target	target		target	
MDC1	target					target
MECOM				target		target
MEST				target		target(3)
MIA				target	target	
MMP1	target	target	target	target	target	target
MMP13			target		target	
MSH2	target					target
MTMR6	target					target(4)
MYB				target	target	
MYCN				target	target	
MYL12B			target		target	
MYL9			target			target(3)
NCALD				target		target(3)
NID1	target				target	
OAT			target		target	
ODC1			target		target	
PARM1		target				target
PCBP1	target	target	target	target	target likely(5)	target
PCCA			target			target
PCOLCE			target			target
PDGFA			target		target	
PECAM1	target					target
PEX11B	target					target
PHF2	target					target
PHF3	target					target
PHYH			target			target
PIP4K2B		target			target	
PKIG			target			target
PKN1	target		target			target
PLEK			target			target(4)
PLSCR1		target	target		target	
PLXNC1	target					target
POSTN	target				target	
PPP1R15A	target				target	

PPT1		target	target			target(3)
PRAF2				target		target(3)
PRRX1	target					target(3)
PRSS23	target				target	
PTX3	target	target	target	target	target	target
RABGAP1	target					target
RAC2		target	target		target	
RAPGEF2	target					target
RARB	target					target(4)
RBCK1			target		target(3)	
RNASE1			target			target
RNASE4				target		target
RNASE6			target			target
S100A2	target	target	target	target	target	target likely
S100A7			target		target	
SACM1L	target					target(3)
SELE	target	target	target	target	target	target
SELENBP1		target				target
SELL			target			target
SEPP1				target		target
SEPT8		target			target	
SEPW1			target		target	
SFN	target	target	target	target	target	target likely
SFPQ		target			target	
SFRP1				target	target	
SGCE			target	target		target
SH3BGRL	target					target
SIK1	target	target			target	
SLA			target			target
SLC35A1	target					target
SLC39A8			target			target
SLC6A8			target		target	
SMARCA2	target					target
SMPD1	target					target(3)
SNX2	target					target
SPARCL1	target					target
SPP1	target	target		target	target	target(3)
SREK1	target					target
SRGN			target			target
ST13			target		target	
STMN1		target	target		target	
TALDO1			target		target	
TCEAL1			target			target
TCN1				target		target
TGFB3	target				target	
TMEM109	target			target	target	target likely(6)
TMEM123	target				target	
TMSB15A				target	target	
TOP2A			target		target likely	
TP63	target	target	target	target	target validated(3)	target(3)
TPM1	target	target	target	target	target validated	target likely(3)

TRA2B		target			target	
TRIM16			target		target	
TRIM44	target					target
TRO		target			target	
TRRAP			target			target
TSPYL4	target					target(3)
TUBA1A		target				target
UBE2J1			target			target(3)
UBL3			target			target
UTP14C	target					target
VGLL4	target				target	
VWF	target					target
WWTR1	target				target	
XK			target			target
YTHDC1	target					target(4)
ZNF451	target					target
ZNF638	target					target
