# Supplementary material for: Unraveling Regulatory Programs for NF-kappaB, p53 and MicroRNAs in Head and Neck Squamous Cell Carcinoma
Source: PLoS One. 2013 Sep 19;8(9):e73656. doi: 10.1371/journal.pone.0073656 (PMC3777940; doi:10.1371/journal.pone.0073656)
Supplement: Text S1 — Binding data of TFs and miRNAs. (PDF) [file pone.0073656.s007.pdf]

Supplementary Text S1

<u>TF binding data</u>

NF-κB subunits RelA, NFκB1 and cRel binding data and NF-κB known targets were extracted from available NF-κB websites: 1) http://www.bu.edu/nf-kb/gene-resources/target-genes/, 2) http://rulai.cshl.edu/cgi-bin/TRED/tred.cgi?process=home , and 3) http://bioinfo.lifl.fr/NF-KB/.
p53 binding data and target genes were curated from previous publications [1,2,3,4,5,6,7,8,9,10,11,12] and website (http://rulai.cshl.edu/cgi-bin/TRED/tred.cgi?process=home).
AP1 binding data was gained from website (http://rulai.cshl.edu/cgi-bin/TRED/tred.cgi?process=home) and literature mining.
Binding data of four TFs (CEBPB, EGR1, STAT3, and SP1) were extracted from database of http://www.broadinstitute.org/gsea/msigdb/. This website provides TF target genes containing conserved binding motifs among human, mouse, rat and dog.

<u>miRNA binding data</u>

Two miRNA (mir21 and mir34ac) binding data were extracted from database of http://www.broadinstitute.org/gsea/msigdb. We also included known binding target genes of mir21 and mir34ac according to miRNA target databases TarBase [13] and mir2disease [14].

<u>List of TF and miRNA binding target genes</u>

1): RelA

IL1R2 REL SERPINE1 TNC BMP4 TNIP1 NFKBIE JUNB AGER ALOX12B APOC3 B2M BCL2 BCL2L1 BHMT BLR1 BM2 CARD15 CCL11 CCL15 CCL19 CCL2 CCL20 CCL23 CCL5 CCR5 CD74 CDKN1A COL1A2 COX2 CR2 CSF1 CSF2 CSF3 CXCR5 DEFB4 DIO2 DNASE1L2 ELF3 F3 FASLG GSTP1 GZMB HBZ HLA-A HLA-B ICAM1 IER3 IFNB1 IFNG IGHG3 IKBKE IL12B IL1A IL1B IL1RN IL28RA IL2RA IL6 IL8 IRF4 IRF7 ITGA5 KLK3 LAMB3 LMP2 MEFV MMP9 MYC MYC NFKB1 NFKB2 NFKBIA NOD2 NOS1 NOS2 NQO1 NR4A2 PDGFB PLA2G4A PLAU PPAP2A PSMA2 PSMB9 PTGS2 PTK2 PTX3 RELB S100A6 SCNN1A SELE SERPINB9 SPP1 STAT4 STAT5A TAP1 TNF TNFRSF10B TP53 VCAM1

2): cRel

JUNB ALOX12B ASS1 CCL2 CCL4 CD74 CDKN1A CR2 CSF2 F3 HIV-1 HLA-A HLA-B IER3 IFNB1 IGHG4 IL12B IL1B IL2 IL2RA IL6 IL8 IRF4 NFKBIA PMAIP1 PLAU TNF BBC3

3): NFκB1

AGER ALOX12B APOC3 B2M BCL2 BCL2L1 BHMT BLR1 BM2 CARD15 CCL11 CCL15 CCL19 CCL2 CCL20 CCL23 CCL3 CCL5 CCR5 CD3G CD40LG CD48 CD74 COL1A2 COX2 CR2 CRP CSF2 CXCR5 DEFB4 DNASE1L2 ELF3 FAS FASLG GNLY GSTP1 GZMB HBZ HIV-1 HLA-A HLA-B ICAM1 IER3 IFNB1 IFNG IGHG3 IL12B IL1A IL1B IL1RN IL2 IL28RA IL2RA IL6 IL8 IRF4 IRF7 ITGA5 JUNB KLK3 LAMB3 LMP2 MIRLET7A3 MMP1 MYC NFKB1 NFKB2 NFKBIA NFKBIE NOD2 NOS1 NOS2 NR4A2 PDGFB PLA2G4A PPAP2A PSMB9 PTGS2 PTX3 RELB SELE SELP SERPINB9 SERPINE1 SPP1 STAT4 STAT5A TAP1 TNC TNF TNFRSF6 TNFSF5 TNIP1 TP53 TP63 VCAM1

4): p53

ADARB1 APAF1 ARHGEF7 ATF3 BAX BBC3 BDKRB2 BID BTG2 C12orf5 C1orf183 CARD16 CASP1 CASP6 CCNG2 CDKN1A CEP57 CTSD DCC1 DDB2 DDIT4 DKK1 DSC3 DUSP1 DUSP14 DUSP5 EDN2 EGFR ENC1 EOMES EPHA2 EVPL FAS FDXR FLT1 FMOD FOS GADD45A GDF15 GJA7 GML GPX1 ID2B IER5 IGFBP3 ITGA3 LITAF LRDD MAD1L1 MAEA MDM2 MLH1 MMP2 NDRG1 NINJ1 NUFIP2 P53AIP1 PA26 PAG608 PCAF PCBP4 PCNA PDGFC PERP PHLDA3 PLAGL1 PLK2 PLK3 PMAIP1 PMS2 PPM1D PPM1J PRKAB1 PROCR PRODH PTEN PTGES PTK2 RPS27L RRM2B RTP S100A2 SAA1 SCARA3 SCGB1D2 SEI1 SEMA3B SERPINB5 SERPINE1 SERPINF1 SERTAD1 SESN1 SESN2 SFN SIVA SNK SOD2 TGFA THBS1 TNFRSF10B TNFRSF10C TNFRSF10D TOB1 TP53AIP1 TP53I11 TP53I3 TP53INP1 TP63 TP73 TRAF4 UBTD1 XPC XRCC5 ZMAT3

5): AP1

ALDOA BDKRB1 CCL2 CCL5 CD40L CD209 CHGA F3 FGF2 FOS GJA1 GJB2 HLA-DRA HSPA4 ICAM1 IFNB1 IL2 IL5 IL6 IL8 IL9 ITGAX IVL JUN KRT16 MMP3 MT1A NOL3 NPPA NPY NQO1 NTS PENK PLAT POLA1 SELE SERPINB2 SFTPD SPRR1A SPRR1B TFPI2 TGFB1 TIMP1 TIMP3 TNF TNFAIP6 TNFRSF9 TP53 TSHB VCAM1 VIM VIP

6): STAT3

AKT1 FOS IL10 BCL2L1 PRF1 ICAM1 APBA1 ASXL1 BTBD1 C11ORF79 C14ORF179 C5ORF41 CCL2 CISH CLDN5 ELMO1 GEN1 HNRPR MAFF PROS1 SERPING1 SLC38A5 TRAF4 UBR1 VIP ZNF228 ACCN1 ACCN4 ADM2 AP1S2 AP2B1 APG3 ARF3 ARHGAP8 ARL6IP6 ARX BCL7A BMI1 BMP4 BNC1 C10ORF77 C17ORF27 C1ORF16 CA10 CALU CAPZA1 CENTD1 CHRM1 COL4A3BP CPA4 CPLX2 CRTAC1 CTGF DD5 DDIT3 EGR1 EGR3 EIF4E EIF4G1 EIF5A EPHA7 FBN2 FBXL3A DERL2 FLJ14103 FLJ14494 FLJ33387 FLJ45530 FLRT1 FOSB FUT8 GABRB1 GPC3 GPHN GRIN2D HEYL HOXB13 HOXB4 HOXB9 HOXC4 HOXC6 HS6ST3 IL18BP IRF1 IRX5 KAZALD1 KCNH3 KCNN2 KCNN3 KIAA0913 KIRREL3 KPNB1 LOC124245 LRP2 LTA LTBP1 LU MAML1 MATN4 MBD6 MEIS2 MGC13053 C17ORF91 MGC15677 MIG12 MIS12 MLL MLLT7 MNT MOBKL2C MTMR14 MYT1 NAPB NAV2 NCAM1 NCOA5 NDST2 NELL2 NFAM1 NR1D1 NR4A1 NXF OAZIN PAPD1 PCBP4 PGF PPFIA2 PR1 PTMS RBPSUH RBPSUHL REM2 RGS3 RIMS1 RIS1 RND1 RSPO2 S100A14 SCUBE3 SDC1 SENP3 SET SHOX2 SLC35A5 SLCO5A1 SLICK SOCS1 SP6 SPON1 SPTBN2 ST7L SV2B TAO1 TCF7L2 TJP4 TM9SF1 TMEM23 TNFSF18 TNRC6 TRIB2 TRIP10 TSC22D4 UBE4B UBTF UPK2 VCL VSNL1 WDR81 WEE1 WNT4 YY1 ZBTB11 ZBTB9 ZFYVE9 ZHX2 ZNF151 ZNF161 ZNF342 ZNF46

7): EGR1

A2LP ACE ADSS AKAP12 ANKH ANKS1 AP1G1 AP3S1 APG12L APLP2 APP ARF3 ARL1 ATE1 ATP1B2 BAHD1 BITE BRD2 BRODL C10ORF4 C11ORF13 C11ORF35 C14ORF4 C18ORF10 C19ORF26 C1ORF66 C20ORF67 C6ORF166 C9ORF37 C9ORF41 C9ORF42 C9ORF72 CACNA1A

CACNA1E CAMK2A CBLN1 CCDC45 CD28 CD40L CDH2 CDKN2C CGGBP1 CLPTM1 CLSTN1 CLTC CMAS CNN2 CNNM4 CNOT6L CORO1C CREB3L1 CSMD3 CTCF DDX5 DDX6 DLC2 DLL4 DLX4 DNAJB5 DNCL1 DNCLI1 DVL2 EFEMP2 EGLN2 EGR1 EGR3 EIF5A ELL EPHB1 ERF ETV4 FAF1 FAM122B FBXL11 FEV FLJ10747 FLJ13197 FLJ30213 FOXP2 FUS FZD1 GCN5L2 GIT1 GLRA3 GLTSCR2 GNB2 GPHN GPR132 GRB2 GRIN1 GSH1 HAS1 HCFC1R1 HCN4 HHEX HMGA1 HMGN1 HMGN2 HNRPDL HOXA2 HOXA5 HOXA7 HOXB8 HR HRD1 HRK HSPB9 HYAL2 ICAM5 IDN3 IL2 IL2RB IMP-1 IMPDH1 ING1L ISG20L2 ITGB8 IWS1 KCNAB3 KCNB1 KCNB2 KCNC1 KCND2 KCNH5 KCNN2 KCNQ5 KCNS2 KIAA1539 KIAA1972 KLF16 KLF3 KLHL10 KRCC1 KREMEN2 LEF1 LOC112476 LOC387882 LRFN5 LYPLA2 MAN2A2 MAP3K4 MASA MATR3 MEF2C MGC15716 MGC4268 MGLL MIG-6 MNT MORF4L2 MRC2 MSI1 MSL2 MY048 MYB MYST2 NAB2 NACSIN NDRG1 NDUFA4L2 NEUROD2 NFYC NIP30 NLK NRGN NRKNSE2 NT5C3L NUMBL NXF ORC4L OTX1 PACSIN2 PAFAH1B1 PARP6 PBX1 PCDH17 PCOLCE PCSK2 PCTK2 PDCD8 PDGFB PITPN PJA1 PLEKHH3 PLOD3 POR PORCN PPARGC1B PRKACA PRKAG1 PRPF3 PSK-1 PTCH PTEN PTMS PTPN7 QRICH1 RAB26 RAB39 RALGPS2 RAPGEFL1 RASGEF1A RBBP6 RELB REXO2 RFX4 RHOA RHOB RNF24 RPS6KA3 RPUSD4 RUNX1 SARM1 SCN8A SDCCAG8 SEC14L1 SEPT15 SERTAD1 SF1 SFRS10 SH3BP1 SH3GL3 SH3KBP1 SHF SIK2 SLC22A17 SLC25A23 SLC25A38 SLC9A7 SMARCA5 SMYD5 SNIP SOCS5 SOX12 SP1 SPATA11 SPRED1 SPTB SREBF1 SREBF2 SSTR3 SYN1 TAF11 TBC1D10 TBX3 TCTA TFAP2C TFE3 TGFB1 THOC6 TIP120A TLE3 TLE4 TNF TNFRSF12A TORC2 TP53 TPBG TPM4 TRIB1 TTC9C TUG1 UBASH3B UBTF UBXD3 UCHL3 USP1 VAMP2 VEGF VGF VTN WDR44 WDR48 XK XPR1 YWHAE ZBTB5 ZFPM1 ZNF198 ZNF232 ZNF827 ZNHIT1

8): CEBPB

ABCA6 ACAA2 ACE2 ACLY ACSL5 ACVR2 ADAMTS5 ADRB2 AGC1 AKR1D1 ALB ALDH1A2 ALDOA ANGPT1 ANKRD11 AP1G1 AP1S2 APC AQP9 ARF6 ARID1B ARNT ARPP-21

ARRDC3 ASAH2 ASCL2 ASGR1 ATAD2 ATP13A4 B4GALT1
BAHD1 BAZ1A BCL11A BCL6 BDKRB1 BDNF BHC80 BMF
BMX BNC2 BNIP3L C10ORF119 C10ORF140 C10ORF69
C11ORF8 C12ORF28 C14ORF116 C14ORF43 C14ORF58
C1ORF122 C1QL1 C1S C20ORF112 C20ORF17 C20ORF177
C20ORF32 C20ORF4 C21ORF25 C3 C5ORF13 C5ORF6
C6ORF1 C9ORF19 C9ORF52 CACNB1 CACNB2 CAPN6 CASK
CASQ1 CBX4 CCDC100 CCDC75 CCL3 CCL5 CCND2
CCNL1 CDC42EP3 CDKL5 CDKN1B CEBPB CEPT1 CFL2
CHAC1 CHD2 CHEK2 CHRM1 CHST9 CIAS1 CIP29 CITED1
CITED4 CKAP4 CLDN10 CLN5 CMT4B2 CNOT1 COL25A1
COL4A3 COL4A4 CP CPNE4 CRIM1 CRTC2 CSMD3 CSNK1E
CSPG2 CSRP1 CTNNAL1 CUEDC1 CXORF15 CXORF26
CYP24A1 D1S155E DBH DCN DDIT3 DDR2 DDX47 DLG2
DLX1 DMD DOCK3 DREV1 DSC1 DSCR1 DSPP DUSP1
DYRK1A DYRK3 ECHDC2 EDG8 EDN2 EFNA5 EHF EIF4A1
EIF4A2 ELAVL2 ELMO3 EP300 EPHA7 EPHB6 ERBB4 ERF
ERRFI1 ESR1 ETF1 ETV1 ETV6 F9 FABP4 FAM5B
FAM70A FAM91A1 FAP FBXL14 FBXW7 FCER1G FGA FGB FGF14
FGF9 FHIT FIGN FLJ12903 FLJ20184 FLJ22457
FLJ34154 FLJ34690 FLJ40536 FLJ40869 FLJ43093
FLJ44048 FLOT2 FMO2 FOS FOXP1 FOXP2 FOXP3 FRMD5
FST G0S2 G10 GABRA3 GARS GLP2R GOT1 GPR3 GPR85
GPR86 GPR97 GPX1 GRM7 GSR GYG GYS1 H2AFJ
H2AFZ H3F3B HAVCR1 HDCMA18P HEXIM2 HIST1H1C HNRPR
HOXA5 HOXC10 HOXC13 HOXC4 HSPC129 HTPAP ID3 IDH1
IGFBP5 IHPK2 IL10 IL19 IL1B IL1F10 IL1RAPL2 IL23A IL27
IL6 IL8 IMPDH1 IRF5 IRLB ITGA11 ITGA5 ITK ITPR3
JMJD2A KCNE3 KCNH7 KCNJ13 KCNJ2 KIAA1737 KLF5
KLHL6 KLHL7 KLK9 KPNA3 KRT23 KRT25A LAF4 LEMD2
LEP LGALS12 LMO4 LOC199675 LOC222171 LOC283514 LOC56901
LOC92799 LPO LRFN4 LRP1 LRRTM3 LUZP1 LYPD1 MAGED2
MAIL MAP2K3 MAP2K6 MAP3K3 MAP4K4 MAPK14 MARCKS MBIP
MBNL1 ME3 MEFV MEOX2 MGC14376 MGC50372 MITF MLR2
MLSTD2 MMP27 MNAB MOSPD2 MPP6 MRC2 MREG MRVLDC1
MSH5 MTF1 MTF2 MTSS1 MYBPC1 MYH3 MYH4 MYH7
MYO1C NADK NAP5 NAT9 NDRG1 NEK6 NFATC4 NFE2L2
NFIL3 NFKBIA NFKBIE NHLH1 NRP2 ODC-P ONECUT2

OTOP3 P2RY1 P2RY4 P8 PAFAH2 PANK3 PAX8 PC326 PCDH7 PCDH8 PCDHGC3 PCF11 PCK2 PCTP PDAP1 PDCL PDE1B PDE3B PDE4D PDGFB PDGFC PDGFRA PDGFRL PER1 PFN2 PHF15 PHF16 PHLDB1 PHOSPHO1 PHOX2B PIK3R1 PIM1 PIP5K1A PITX2 PKNOX2 PLA2G2E PLA2G4A PLCB1 PLCB2 POLG PP1665 PPARG PPL PPM1A PPM1B PPP1CB PPP1R3D PRDM16 PRDX3 PRDX4 PRG4 PRKCG PRKWNK2 PRLR PTCH PTGIR PTGS2 PTPN12 PTPNS1 RAB2 RAB3IP RAD23B RARB RASAL2 RBPMS RCOR1 RCP REXO2 RFX4 RG9MTD2 RGN RGR RGS3 RHOB RIN3 RNF127 RNPC2 RORA RPA3 RPS21 RRBP1 RS1 RSN RSPO2 RUVBL2 S100A9 SAA1 SAA2 SARS SCYL2 SDPR SEPHS2 SERPINA7 SERPINE1 SERPINF1 SERTAD4 SFTPD SFXN4 SHFM3 SLC12A1 SLC19A3 SLC25A12 SLC25A35 SLC39A13 SLC6A4 SLC7A11 SLIT3 SMAD1 SMAD6 SMARCA1 SMARCA2 SMARCAD1 SMC6L1 SOBP SOCS2 SOX10 SOX5 SP8 SPAG9 SPIB SPRED1 SPRR1B SPRY4 SPTLC2 SRPK1 ST18 STAT3 STC1 STC2 STEAP2 STEAP4 STK39 STMN1 STRN STX18 SULF1 SYNCRIP SYNJ1 TAC1 TAFA1 TBC1D10B TBL1X TBL1Y TBR1 TCF12 TDRD4 TFDP2 TFE3 TGFB3 TGIF THRA TM4SF9 TMEM104 TMPRSS4 TNF TNFAIP6 TNFSF13B TNFSF14 TNNC2 TOB1 TOP1 TP63 TPM2 TRA1 TRALPUSH TRIB1 TRPM1 TSGA14 TUBA3 TUBB6 UBE2E2 UBQLN1 UGP2 UNQ689 UNQ698 USP9X VAMP3 VIT VNN3 VPS13C WDR81 WHSC1L1 WNT10B WNT5A WNT6 YIPF7 YRDC ZADH2 ZFHX1B ZFP36L1 ZFYVE9 ZIC1 ZIC4 ZNF217 ZNF238 ZNF288 ZNF537

9): SP1

A2BP1 ABCC1 ABHD1 ACBD5 ACCN2 ACE ACLY ACVR2 ADAM10 ADAM15 ADAM17 ADCK1 ADRB1 ADSS AF1Q AF5Q31 AGBL5 AGER AHNAK AKT2 ALOX12 ALS2 ALS2CR13 ANKMY2 ANKRD12 ANKRD25 ANKS1 ANP32A AP1GBP1 AP3M2 APLP1 APLP2 ARCH ARFIP1 ARHGEF1 ARHGEF19 ARHN ARID1A ARL3 ARPC4 ASB7 ASC1P100 ASCL2 ATP2A2 B4GALT2 BAD BAHD1 BCL11B BCL2 BCL2L2 BCL3 BCL6 BCL6B BCL7C BCL9L BDNF BHC80 BHLHB3 BIRC5 BMI1 BMPR2 BRI3BP BRMS1L BRUNOL6 BTBD14B BZW2 C11ORF13 C11ORF30 C11ORF4 C11ORF56 C11ORF79

C12ORF22 C13ORF25 C14ORF50 C14ORF58 C17ORF28
C17ORF31 C17ORF61 C1ORF122 C1ORF172 C1ORF27
C1ORF43 C20ORF104 C20ORF133 C20ORF177 C20ORF23
C20ORF67 C2ORF21 C3F C6ORF134 C6ORF49 C9ORF37
CACNA1A CACNA1B CACNA1D CALM3 CAMK2G CAMKK1
CASQ1 CBLN1 CCDC136 CCL15 CCND2 CCNE1 CD209
CD3EAP CDC25A CDC37 CDC40 CDCA7 CDH2 CDH24 CDH3
CDK5R1 CDKL5 CDKN1A CDW92 CEACAM5 CEBPE CENTD2
CGGBP1 CHAT CHD6 CHKA CHPF CITED2 CKAP1 CLDN11
CLPTM1 CLSTN1 CNNM4 COL12A1 COL13A1 COL4A2 COLIA1
COPS5 CORO1C CPD CREB1 CRY1 CRYAB CS CSNK1D
CSPG4 CTTNBP2NL CXCL1 CXCL12 CXCL5 CYP26B1 D4ST1
DACH1 DDAH2 DDB1 DDX6 DES DET1 DFNB31 DGKA DHH
DIPA DKFZP434E2321 DKFZP586B1621 DLC2 DLL4 DLX3
DMTF1 DNAH9 DNAJC4 DNCL1 DNM3 DNMT3A DPAGT1 DRAP1
DUOX1 DUSP15 ECE1 ED1 EDG8 EDN3 EFEMP2 EFNA3
EFNB3 EGLN2 EIF2C1 EIF5A ELAC2 ELAVL3 ELK1 ELKS
ELL EML3 EN1 ENAH EPB41 EPHA1 EPLIN EPN2 EPO
EPS15 EPS8L2 ERBB3 ERG ERO1LB ET ETV5 FAF1 FAM100B
FAPP2 FASLG FBS1 FBXL11 FBXL19 FBXO3 FBXO34 FBXO36
FDX1 FEV FGF11 FGF12 FGF5 FKBP14 FKBP8 FLII FLJ12439
FLJ12529 FLJ13855 FLJ14069 FLJ20920 FLJ23221
FLJ23342 FLJ23436 FLJ23861 FLJ30058 FLJ31842
FLJ32549 FLJ39653 FLJ40342 FLJ44313 FLJ90811 FNTA
FOSB FOXP4 FRMD5 FRS3 FSTL5 FURIN FUS FUT8 FXR2
FZD2 G22P1 GABARAP GABARAPL2 GAD1 GALE GALNS
GAP43 GAS GAS7 GASP GBF1 GCN5L2 GFRA1 GGA1 GGN
GJA1 GJB6 GK GLI GLTP GM117 GMIP GMRP-1 GNA11
GNL1 GOLGA3 GPR3 GRB2 GRIN2A GRIPAP1 GSC GYLTL1B
GYPC HAP1 HBZ HCFC1R1 HCN4 HDAC6 HES7 HIPK1
HMGA1 HMGN2 HNF4A HNRPA2B1 HNRPDL HNRPUL1 HOXA3
HOXB7 HOXC13 HPN HRAS HRH3 HRMT1L2 HS2ST1
HSD11B2 HSD17B1 HSPB1 HSPB2 HSPB9 HTATIP HTR1A
HTR7 HUMAGCGB HYAL2 IDH3A IDH3G IGF2 IGF2BP1
IGSF4 IGSF4D INA INCA1 ING1L ING3 INPPL1 INSR
IQGAP1 IRF2BP1 IRX3 ITGAM ITGAV JMJD1A JUB JUN KCNB1
KCNC3 KCND1 KCND2 KCNE1L KCNIP2 KCNMA1 KCNN2 KCNQ4
KIAA0276 KIAA0528 KIAA0922 KIAA1259 KIF1C KIT

KLF13 KLF16 KLF2 KLF5 KLF7 KLHL7 KLK8 KNSL8
KPNB1 KRT16 KRT18 KTN1 LASP1 LCAT LDB1 LHX3
LLGL2 LMO6 LOC114926 LOC115704 LOC129285 LOC143425
LOC149420 LOC196549 LOC253982 LOC283932 LOC56901
LOC57149 LOC84661 LOC90557 LOC91056 LOXL4 LPPR2
LRP1 LRRC4 LRRC5 LRRC8E LTC4S LYL1 LZTS2 MAGED1
MAGED2MAP2K7 MAP3K11 MAP3K6 MAP3K7 MAP4K2 MAPK7 MASA
MAZ MDS1 ME3MGC14376 MGC35138 MGC9564 MIP MLP
MN1 MNT MPP5 MPRIP MRPL2 MSPMTF1 MTMR3
MTSS1 MUC1 MXD4 MYADM MYLIP MYO1C MYOHD1
NAGK NASP NAV1 NCE2 NCOA2 NCOA6 NDRG1 NDST2
NEF3 NET1 NFAT5 NFE2L1 NFKB1 NFKBIA NFYB NFYC
NIP NLKNOL3 NOL4 NOTCH3NPYNR2F2 NRAS NRG2 NTF5
NTRK3 NUB1 NUBP2 NUFIP2 NUMBL NXFNXPH3 NXPH4 OAZ2
ODC1 OLIG2 ONECUT1 OPRM1 OSBP OSBP2 OSBPL9 OTX1
P5326 PACS1 PAK1 PAK4 PARD6A PAX2 PCF11 PDE4C
PDGFB PDLIM7 PER1 PEX14 PGFPGM2L1 PGRPHCA PHF15
PHF23 PHLDA3 PHOX2A PIAS1 PIAS3 PIGN PIGV PIGW
PIK4CB PIP5K2BPITPN PITPNM1 PKP3 PLCD1 PLEKHB1
PLEKHM1 PMM1 PNUTL2 PODN POLR2I POLR3E PORCN
POU2F1 POU3F2 POU5F1 PPARGC1B PPFIA2 PPM1J PPP1R3D
PPP2R3A PPRC1 PRKRA PROSTEIN PRR3 PRSS12 PSEN1
PSMC6 PTCH2 PTDSR PTHLH PTK7 PTOV1 PTPN2 PTPRJ
PVRL2 RAB10 RAB26 RAB2B RAB35 RAB9A RABEP2 RAM2
RANBP10 RAP1GDS1 RARA RBBP4 RBBP7 RBM4B RCOR2
RDH10 REL RELA RELB REM1 RFPRGL3 RHBDL4 RHOG
RING1 RKHD3 RNF110 RNF24 ROBO3 ROD1 ROM1 RPL7
RTF1 RTN3 SCA1 SCARF1 SCARF2 SCYL1 SEC24C SEC63
SENP1 SEPT15 SERPINB2 SERPINE1 SESN2 SEZ6L2 SFRS2
SFRS7 SFXN2 SH2BP1 SH2D3C SH3BP5 SH3KBP1 SHFM3
SHMT1 SIAT7C SIPA1 SIX4SLBP SLC18A3SLC1A2 SLC24A6
SLC2A1 SLC30A3SLC35F5SLC7A10SLC9A6 SLITRK4 SLITRK5 SMAP1
SMARCA1 SMARCA5 SMARCD1 SMARCE1 SMBP SMCR7
SMOC1 SNAG1 SNAPAP SNCB SNX2 SOX12 SOX2 SP3 SP4
SPAG9 SPATA6 SPEC1 SPG4 SPN SRR SSB3 SSR4 STMN1
SUPT6H SUV39H2 SWAP70 SYNCRIPTADA3L TAF11 TAF6
TAGLN TAGLN2 TAL1 TAPT1 TBX2 TBX3 TBX5 TCEAL1
TCF12 TCF4 TDE2L TEAD2 TEF TERT TGFB1 TGM4

THOC6 THRA TIEG2 TIGD4 TIMELESS TIMP1 TIMP3 TIPRL TITF1 TLX2 TMEFF1 TMEM150 TMEM24 TMP21 TMUB2 TNF TNFAIP3 TNRC5 TPI1 TPM4 TPR TRIM28 TRMT1 TRPC4AP TRPM4 TSNAXIP1 TTC11 UBE2D3 UBE2O UGP2 ULK1 UNC13B UPF2 UST UTP18 VAMP2 VASP VCAM1 VEGFA VGLL2 VKORC1L1 VLDLR VPRBP WASF1 WDFY2 WDR21 WDR81 WHSC1L1 WINS1 WNT1 WNT2 WNT2B WNT5A WWP1 XPR1 YRDC ZBTB5 ZFP36 ZFP91 ZFYVE16 ZNF161 ZNF282 ZNF403 ZNF414 ZNF512 ZNF524 ZNF553 ZNF687 ZNF703

10): mir21

ACBD5 ALX1 ANP32A AP1AR ARHGAP24 ARID1A ASPN BAHD1 BASP1 BCL2 BMPR2 BNC2 BOLL BRD1 BTBD3 BTG2 C12orf12 C17orf39 CASKIN1 CBX4 CCL1 CDC25A CHD7 CNTFR CPEB3 CREBL2 CREBRF CRIM1 DAZL DERL1 DNAJC16 EHD1 EIF2C2 ELF2 EPHA4 FAM193B FASL FASLG FBXO11 FCHO2 FLJ35409 FUBP1 GATAD2B GLCCI1 GPR64 HNRNPK ING3 JAG1 JPH1 KCNA3 KLHDC5 KRIT1 LEMD3 LRRFIP1 MARCKS MATN2 MBNL1 MIR137HG MPRIP MRPL9 MTAP NAA50 NCAPG NF2 NFIB NTF3 OSR1 PAN3 PBRM1 PCBP1 PCBP2 PCDH17 PCSK6 PDCD4 PELI1 PER2 PHF14 PIK3R1 PITX2 PLAG1 PLEKHA1 PLOD3 PPARA PPP1R3A PPP3CA PSRC1 PTEN PURB RAB11A RASGRP1 RECK REST RHOB RMND5A RNF103 RNF111 RNFT1 RPS6KA3 RPS7 RTN4 SATB1 SCML2 SERPINB5 SFRS3 SFRS8 SFSWAP SKI SMAD7 SMARCA4 SOX2 SOX5 SOX6 SPRY1 SPRY2 SRSF3 SSFA2 STAG2 STAT3 STK40 TAGAP TGFBI TGFBR2 THRB TIMP3 TM9SF3 TNRC6B TP53 TP63 TPM1 TRAPPC8 TRPM7 UBE2D3 UBE4A UBR3 WFS1 WIBG WNT1 WWP1 XKR6 YAP1 YOD1 ZCCHC3 ZNF367 ZNF654 ZNF704

11): mir34ac

AADACL1 ABCF3 ABR ACBD3 ACCN1 ACSL1 ACSL4 ACTR1A AKAP1 AKAP6 ALCAM ALDOA ANK2 AP2S1 APH1A ARHGAP1 ARHGAP26 ARID4B ASB1 ATG4B ATP5S ATXN2L AXL B3GAT3 B4GALT2 BAZ2A BCL2 BCL6 BCL9L BEST1 BIRC3 BRPF3 BTBD11 C14ORF28 C14ORF43 C1ORF116 C1ORF9

C20ORF24 C22ORF5 C8ORF13 CA7 CACNB1 CACNB3 CALCR CAMTA1 CAPN6 CASP2 CBFA2T3 CCBL1 CCND1 CCNE2 CD44 CDK6 CDKN2C CEBPB CHMP7 CNOT4 CNOT6 CNTN2 CNTNAP1 CNTNAP2 COL12A1 COPS7B CORO1C CPEB3 CPLX2 CREB5 CRHR1 CSF1R CSNK1G1 CSNK1G3 CTNND2 CUEDC1 CUGBP2 DAAM1 DBC1 DCX DDX17 DGKZ DIXDC1 DLL1 DNAJC16 DPP3 DPYSL4 E2F3 E2F5 EI24 EIF2C4 ELMOD1 EML5 ERGIC1 ESRRA EVI5L F2RL2 FAM123B FAM126B FAM70A FAM76A FGD6 FIGN FNDC8 FOXG1B FOXJ2 FOXP1 FRMD4A FUT8 GALT GDAP1L1 GLCE GMFB GPHB5 GPR64 GPR85 GRID1 GRK6 GRM7 HCN3 HDAC1 HNF4A HTR2C ILKAP IQGAP3 ITSN1 JAG1 JAKMIP1 KCNH2 KIAA0552 KIAA0789 KIAA0828 KIAA1217 KITLG KLF12 KLF4 KRT40 LDHA LEF1 LGR4 LIMD2 LMAN2L LOC284296 LOXL3 LPHN1 LRRC55 LYST MAP1A MAP2K1 MAPT MARCH5 MET METAP1 MGAT4A MGAT5B MLLT3 MPP2 MSL2L1 MTA2 MYC MYCN MYRIP NAT13 NAV1 NAV3 NETO1 NFE2L1 NOTCH1 NOTCH2 NPTX1 NR4A2 NRIP3 NRXN2 NTNG2 NUMBL OSGIN2 OTX1 PACS1 PDGFRA PEA15 PGM1 PKIA PKP4 PLCB1 PLEKHG5 PLOD1 PNOC POFUT1 POGZ PPFIA1 PPP1CC PPP1R10 PPP1R11 PPP1R14D PPP1R16B PPP2R3A PPP2R5A PRG-4 PTPRM PURB RAB11FIP4 RAB43 RALGDS RALGPS2 RANBP10 RARG RGS17 RIC8B RIMS4 RKHD2 RNF4 RNF41 RPS6KA4 RRAS RTN4RL1 RUTBC1 SAR1A SATB1 SATB2 SBK1 SCN2B SDHC SEC61A1 SEMA4B SEMA4C SEMA4F SEMA5B SEPT3 SERPINE1 SGPP1 SHKBP1 SIDT1 SLC2A13 SLC2A4RG SLC44A2 SLCO3A1 SNW1 SNX15 SOX4 SP2 SPEG SPI1 SPRN SPRY3 SRPR STAC2 STC1 STK35 STRN3 SVOP SYNJ1 SYT1 SYT4 SYVN1 TAF5 TBC1D25 TBL1XR1 TCF12 TFDP2 TGIF2 TMCC3 TMEM109 TMEM55A TMUB2 TNRC4 TNRC6B TOB2 TPD52 TRIM35 TTC19 UBP1 UHRF2 VAMP2 VAT1 VCL VEGFA VEZT WASF1 WBSCR20 WNT1 XPO5 XYLT1 YY1 ZBTB39 ZC3H7B ZCCHC17 ZDHHC17 ZDHHC23 ZER1 ZFHX4 ZMYM4 ZNF207 ZNF281 ZNF282 ZNF3 ZNF644

References:

1. Menendez D, Inga A, Resnick MA (2009) The expanding universe of p53 targets. Nat Rev Cancer 9: 724-737.
2. Riley T, Sontag E, Chen P, Levine A (2008) Transcriptional control of human p53-regulated genes. Nat Rev Mol Cell Biol 9: 402-412.
3. Horvath MM, Wang X, Resnick MA, Bell DA (2007) Divergent evolution of human p53 binding sites: cell cycle versus apoptosis. PLoS Genet 3: e127.
4. Wei CL, Wu Q, Vega VB, Chiu KP, Ng P, et al. (2006) A global map of p53 transcription-factor binding sites in the human genome. Cell 124: 207-219.
5. Harms K, Nozell S, Chen X (2004) The common and distinct target genes of the p53 family transcription factors. Cell Mol Life Sci 61: 822-842.
6. Robinson M, Jiang P, Cui J, Li J, Wang Y, et al. (2003) Global genechip profiling to identify genes responsive to p53-induced growth arrest and apoptosis in human lung carcinoma cells. Cancer Biol Ther 2: 406-415.
7. Nakamura Y (2004) Isolation of p53-target genes and their functional analysis. Cancer Sci 95: 7-11.
8. Polyak K, Xia Y, Zweier JL, Kinzler KW, Vogelstein B (1997) A model for p53-induced apoptosis. Nature 389: 300-305.
9. Somasundaram K (2000) Tumor suppressor p53: regulation and function. Front Biosci 5: D424-437.
10. Vousden KH, Lu X (2002) Live or let die: the cell's response to p53. Nat Rev Cancer 2: 594-604.
11. Qian H, Wang T, Naumovski L, Lopez CD, Brachmann RK (2002) Groups of p53 target genes involved in specific p53 downstream effects cluster into different classes of DNA binding sites. Oncogene 21: 7901-7911.
12. Jegga AG, Inga A, Menendez D, Aronow BJ, Resnick MA (2008) Functional evolution of the p53 regulatory network through its target response elements. Proc Natl Acad Sci U S A 105: 944-949.
13. Vergoulis T, Vlachos IS, Alexiou P, Georgakilas G, Maragkakis M, et al. (2012) TarBase 6.0: capturing the exponential growth of miRNA targets with experimental support. Nucleic Acids Res 40: D222-229.
14. Jiang Q, Wang Y, Hao Y, Juan L, Teng M, et al. (2009) miR2Disease: a manually curated database for microRNA deregulation in human disease. Nucleic Acids Res 37: D98-104.
